# Evaluating alcohol consumption in adolescents and young adults: a meta-analysis of the psychometric properties of measurement instruments

**DOI:** 10.3389/fpsyg.2025.1591800

**Published:** 2026-01-09

**Authors:** Hugo Sinchi-Sinchi, Andrés Ramírez, Luis Burgos-Benavides, Francisco Javier Rodríguez-Díaz, Francisco Javier Herrero Díez

**Affiliations:** 1 Carrera de Psicología, Grupo de Investigación en Psicología Comunitaria y Salud (GIPCS), Pontificia Universidad Católica del Ecuador, Esmeraldas, Ecuador; 2 Carrera de Psicología Clínica, Grupo de Investigación en Neurociencia Clínica Aplicada (GINCA), Universidad Politécnica Salesiana, Cuenca, Ecuador; 3 Departamento de Psicología, Universidad de Oviedo, Oviedo, España

**Keywords:** alcohol, adolescents, psychometric, meta-analysis, young adult

## Abstract

**Introduction:**

Early alcohol consumption is a public health concern among young people in most Western countries. However, psychometric evidence on the tools used to assess alcohol consumption and its associated factors is limited. This meta-analysis aimed to evaluate the overall reliability and measurement quality of instruments assessing alcohol consumption, following standardized methodological frameworks.

**Method:**

The protocol was registered in PROSPERO (ID: CRD4202424533078) and followed PRISMA guidelines for systematic review and meta-analysis. The search was conducted in PubMed, Scopus, PsycINFO, and Web of Science using the PECOS strategy. Methodological characteristics of the studies and the reported psychometric properties of the instruments were documented. Reliability estimates and confidence intervals were analyzed using a restricted maximum likelihood random-effects model. Methodological quality and risk of bias were evaluated based on the COSMIN checklist for systematic reviews of outcome measurement instruments and the Terwee quality criteria, ensuring the inclusion of studies with acceptable internal consistency (α ≥ 0.70) and appropriate model fit (CFI ≥ 0.90; RMSEA ≤ 0.08).

**Results:**

Twenty-seven studies met the inclusion criteria, reporting Cronbach’s alpha coefficients between 0.71 and 0.98. The pooled reliability coefficient was α = 0.88 (SE = 0.0088; 95% CI = 0.86–0.89), indicating high internal consistency. Significant heterogeneity (*I*^2^ = 99.6%) was observed, primarily related to differences in sample size and item number. In addition, according to COSMIN and Terwee assessments the Alcohol Use Disorders Identification Test (AUDIT) and the Brief Young Adult Alcohol Consequences Questionnaire (BYAACQ) demonstrated the strongest psychometric robustness, supported by consistent reliability and validity across cultural contexts.

**Conclusion:**

This meta-analysis provides strong evidence of reliability and adequate structural validity for instruments measuring alcohol consumption in adolescents and young adults. The AUDIT and BYAACQ emerge as the most reliable and valid options, while the CLASS, PRQ, and ACQ-SF-R also represent promising, psychometrically viable alternatives for assessing beliefs, parental norms, and craving associated with alcohol use. These instruments can guide early detection and preventive interventions, supporting more consistent measurement standards in public health and behavioral research.

## 1 Introduction

Alcohol is the most widely consumed psychoactive substance in the world (Gomà-i-Freixanet et al., 2023). In most Western countries, alcohol consumption is a significant public health concern among adolescents and young adults (Smit et al., 2020; Campbell et al., 2022; Stamates et al., 2023). In 2021, approximately 2.3 billion people consumed alcoholic beverages worldwide, and about 283 million people over the age of 15 were diagnosed with alcohol use disorder (World Health Organization, 2021).

In recent decades, reports from Anglo-Saxon countries indicate that the prevalence of alcohol consumption among adolescents and young adults ranges from 20 to 70% (Morales Quintero et al., 2019; Villarosa-Hurlocker et al., 2020; Hummer et al., 2022). The highest prevalence is observed at age 15 (Toner et al., 2019). In 2019, the global per capita alcohol consumption among individuals aged 15 years and older was 5.5 liters of pure alcohol (World Health Organization, 2023). In North America, 78.2% of individuals aged 15 or older reported drinking alcohol in the past year (Dermody et al., 2023).

Early alcohol consumption, widespread normalization, and low risk perception (Sánchez-García et al., 2020) result from culturally ingrained norms in society. The pervasive nature of alcohol consumption underscores the need for early detection (Hadland et al., 2019; Duffy et al., 2023). Alcohol consumption has a profound impact on adolescent and young adult development, education, and social and family relationships. It is also significantly associated with unemployment, low socioeconomic status, traffic accidents, and violence (Klimkiewicz et al., 2021; Voss et al., 2021).

Risk behaviors associated with alcohol consumption among adolescents and young adults generally follow two patterns. The first is continuous and moderate consumption throughout the week without intoxication, commonly referred to as the Mediterranean drinking style (Callinan et al., 2022). The second is characterized by heavy episodic drinking, also known as binge drinking, commonly referred to as the Anglo-Saxon drinking style (Cox et al., 2022).

Several validated and reliable psychometric instruments are available for assessing alcohol consumption (Rodrigues et al., 2021; Delawalla et al., 2023). The Alcohol Use Disorders Identification Test (AUDIT) and its shorter version, the AUDIT-C, are the most widely used psychometric tools for diagnosing alcohol consumption in the general population (Toner et al., 2019). The AUDIT is the most commonly used international instrument (Ohtani et al., 2023) due to its affordability, ease of administration, and rapid application. It has demonstrated strong psychometric reliability for detecting hazardous drinking and dependence in various countries (O'Brien et al., 2020; Klimkiewicz et al., 2021; Duffy et al., 2023).

Unlike Toner et al. (2019), the present meta-analysis broadens the scope by including studies published between 2019 and 2024 and integrates both reliability estimates and model-fit indices (CFI, TLI, RMSEA, SRMR) of psychometric instruments used to assess alcohol consumption and associated factors (Ramírez et al., 2025). Furthermore, it compares instruments that evaluate consumption levels, motives, consequences, and protective factors, thus providing an updated and comprehensive synthesis of psychometric evidence supporting research and clinical applications in adolescent and young adult populations.

The objective of this systematic review and meta-analysis was to evaluate the overall reliability and model fit of psychometric instruments reporting Cronbach’s alpha as a measure of internal consistency, and to identify the most frequently used instruments along with their key psychometric characteristics.

Specific objectives: (1) To describe the methodological and psychometric characteristics of the instruments identified in the systematic review; (2) To analyze the overall and instrument-specific reliability based on Cronbach’s alpha coefficients; (3) To evaluate the overall and instrument-specific model fit using confirmatory indices such as the Comparative Fit Index (CFI), Tucker–Lewis Index (TLI), Root Mean Square Error of Approximation (RMSEA), and Standardized Root Mean Square Residual (SRMR).

### 1.1 Materials and methods

A systematic review and meta-analysis were conducted following the PRISMA Declaration guidelines (Page et al., 2021). The meta-analysis protocol was registered in PROSPERO 2024 (ID: CRD42024533078).

The search terms were defined using the PECOS strategy. Population: Children, adolescents, and young adults up to 25 years old. Exposure: Assessment, diagnosis, or measurement of alcohol consumption. Comparison: Psychometric instruments, questionnaires, scales, and tests used. Outcome: Generalized reliability of psychometric instruments. Study design: Quantitative studies reporting psychometric data of interest.

### 1.2 Inclusion criteria

Studies were included if they met the following criteria: (1) Original quantitative studies assessing alcohol consumption, (2) Studies conducted with children, adolescents, and young adults (up to 25 years old), (3) Studies that used psychometric instruments to assess alcohol consumption, (4) Studies that reported psychometric data and overall reliability of the instrument, (5) Studies with a sample size of at least 10 participants per item of the instrument, (6) Studies published in the last 5 years (2019–2024).

### 1.3 Exclusion criteria

Studies were excluded if they met any of the following criteria: (1) Systematic reviews, meta-analyses, book chapters, theses, conference proceedings, or abstracts, (2) Studies on adult populations over 25 years old, (3) Studies reporting participants with polysubstance use or other drug consumption, (4) Qualitative studies using interviews or surveys, (5) Studies with participants diagnosed with dual pathology or psychiatric disorders, (6) Studies that conducted evaluations exclusively in an online format.

### 1.4 Sources of information

The search was conducted in the following databases: PubMed, Scopus, Web of Science, and PsycINFO. These databases were selected based on their scientific quality and international relevance in health and social sciences. Additionally, the reference lists of included studies were examined to minimize exclusion bias due to database coverage.

### 1.5 Search strategy

The search phrase was developed using a combination of terms frequently used in original research and review studies on alcohol consumption: (“Alcoholism” OR “Alcohol Drinking” OR “Binge Drinking” OR “Underage Drinking”) AND (“Child” OR “Adolescent” OR “Young Adult”) AND (“Assessment” OR “Measure” OR “Measurement” OR “Diagnosis”) AND (“Test” OR “Scale” OR “Questionnaire”). The search terms were adapted to each database (see Supplementary S1).

The decision to limit the search to studies published in the last 5 years was based on a bibliometric analysis of academic production trends in alcohol consumption research. Publication metrics from the four databases indicated a significant decline in studies on this topic since 2019: 779 studies (2019), 679 (2020), 617 (2021), 421 (2022), 392 (2023), and 58 in the first quarter of 2024 (see Supplementary S2).

This decline may be due to shifts in research priorities, particularly the focus on COVID-19 during this period. Therefore, including studies up to 2024 ensures that the findings incorporate reliable and relevant methodological approaches, population trends, and recent developments in the field.

### 1.6 Review strategy

The systematic search was conducted on March 18, 2024. Search records were verified by the principal investigator and a collaborator. After ensuring consistency across the four databases, the records were downloaded in RIS format.

The Rayyan web application (Ouzzani et al., 2016) was used to code the records. Functions such as duplicate detection, inclusion, exclusion, and potential inclusion were applied. Tags were used to document the reasons for selection decisions. All data were stored in the Rayyan system.

A total of 4,412 records were identified. Duplicates were removed using both algorithmic and manual detection, eliminating 1,462 articles due to over 90% content overlap. Based on a rigorous title and keyword screening, 2,760 articles were excluded according to the predetermined inclusion/exclusion criteria. After screening abstracts, 112 additional studies were excluded.

Among the 78 full-text studies assessed, 51 were excluded for the following reasons: Cronbach’s alpha was not reported as a global measure (*n* = 11). Studies did not report psychometric data (*n* = 15). The mean participant age exceeded 25 years (*n* = 10). Full-text access was unavailable (*n* = 4). The study was outside the publication range (*n* = 3). Online-only assessment was performed (*n* = 4). The study was a preliminary investigation (*n* = 1). The instrument used was not a psychometric measurement tool (*n* = 1). The study was based on surveys rather than psychometric instruments (*n* = 1). The study included participants who used non-alcoholic drugs (*n* = 1).

**Figure 1:**
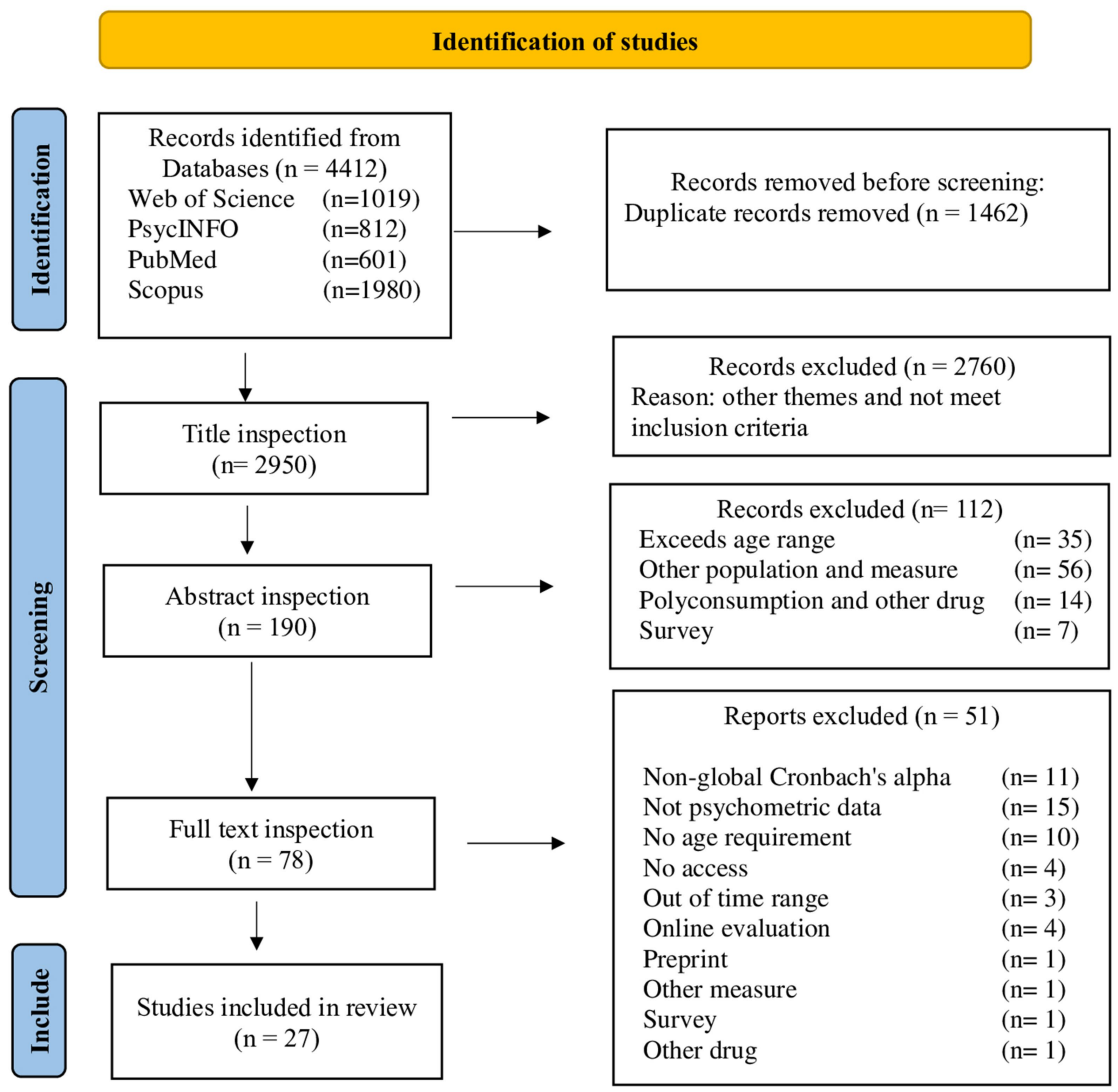
PRISMA flowchart.

The final selection was determined through full-text peer review by the reviewer and a collaborator. Disagreements were resolved through deliberation and consensus. Ultimately, 27 articles were included in the systematic review and meta-analysis (Figure 1).

### 1.7 Data extraction process

Two primary data categories were defined: Methodological characteristics of the included studies and Psychometric properties of the instruments.

The data were extracted according to predefined variables and cross-checked by the reviewer and collaborator. Discrepancies were resolved through comparison and verification.

The following variables were collected: (1) Study characteristics: Author, year of publication, title, journal, quartile ranking, and sample size. (2) Instrument characteristics: Psychometric test, number of items, country, participant age, gender distribution, and prevalence of risky or harmful alcohol consumption.

Psychometric properties: (1), Reliability (Cronbach’s alpha), (2) Goodness-of-fit indices for the structural model, including: Comparative Fit Index (CFI), Root Mean Square Error of Approximation (RMSEA), Tucker-Lewis Index (TLI), Standardized Root Mean Square Residual (SRMR).

### 1.8 Risk of bias and quality assessment

The methodological quality of the included studies and the measurement properties of the instruments were evaluated according to the quality criteria proposed by Terwee et al. (2007), the guidelines of the COSMIN Manual for Systematic Reviews of Outcome Measurement Instruments (Mokkink et al., 2024), and the methodological recommendations of the JBI Manual (Stephenson et al., 2020), specific to psychometric reviews.

Each study was analyzed across the following psychometric quality domains: reliability (internal consistency and temporal stability), structural validity (model fit indices: CFI, TLI, RMSEA, and SRMR), content validity (item representativeness and conceptual clarity), and cross-cultural validity (linguistic adaptation and population relevance). Internal consistency was considered acceptable when Cronbach’s alpha was ≥ 0.70, following Terwee et al. (2007).

Model fit indices were interpreted according to COSMIN guidelines: CFI ≥ 0.90, TLI ≥ 0.90, RMSEA ≤ 0.08, and SRMR ≤ 0.05, indicating good structural model fit. The risk of bias was rated across four levels (very good, adequate, doubtful, or inadequate), considering study design, sample size adequacy (minimum of 10 participants per instrument item), and completeness of reported psychometric information.

The evaluation was independently conducted by two reviewers, with an inter-rater agreement of 96.4%, and discrepancies were resolved by consensus. Detailed results of this assessment, including the scoring of measurement properties and methodological quality following the Terwee criteria and COSMIN risk-of-bias rubric, are provided in Supplementary S3.

Finally, potential sources of heterogeneity were explored through subgroup and sensitivity analyses to distinguish statistical variability from methodological heterogeneity (Harrer et al., 2021).

### 1.9 Statistical analysis

This study employed reliability generalization and meta-analytic approaches to evaluate internal consistency and model fit metrics of alcohol assessment instruments. Cumulative reliability was estimated using Cronbach’s alpha, transformed through the Hakstian–Whalen method to facilitate random-effects modeling (Hakstian and Whalen, 1976).

The studies also reported model fit indices, including the Comparative Fit Index (CFI), the Tucker–Lewis Index (TLI), the Root Mean Square Error of Approximation (RMSEA), and the Standardized Root Mean Square Residual (SRMR). Heterogeneity was assessed using Cochran’s Q, I^2^, H^2^, and τ^2^ statistics (Higgins and Thompson, 2002). Studies deviating from alpha > 0.70 or a 10:1 participant-to-item ratio were excluded using trimming techniques.

Potential publication bias was evaluated through funnel plot asymmetry and Egger’s regression test. The meta-analysis provided a comprehensive synthesis of reliability and validity indicators across diverse applications, allowing for the generalization of psychometric properties in alcohol assessment.

All statistical analyses were conducted using The Jamovi Project (2023) version 2.4.8 and R (Metafor package), version 2023.09.1 + 494.

## 2 Results

A total of 27 scientific articles were included in this systematic review and meta-analysis. These studies report the use of psychometric instruments to assess alcohol consumption in adolescents and young adults. The characteristics of the studies and instruments are described below (Table 1).

**Table 1:** Articles selected for meta-analysis.

No.	Author	Title	Journal	*Q*	Instrument	Country	Age	*n*	Item	Cronbach’s alpha
1	Morales Quintero et al. (2019)	Psychometric properties of the Alcohol Use Disorders Identification Test (AUDIT) in adolescents and young adults from Southern Mexico	Alcohol	2	AUDIT (Saunders et al., 1993)	Mexico	18.16	1,932	10	0.80
2	Miller et al. (2019)	Development and initial validation of the alcohol-induced blackout measure	Addictive behaviors	1	ABOM (Miller et al., 2019)	United States	21.78	350	5	0.91
3	López et al. (2019)	Psychometric properties and factor structure of an Ecuadorian version of the Alcohol Use Disorders Identification Test (AUDIT) in college students	PLOS ONE	1	AUDIT (Saunders et al., 1993)	Ecuador	21.49	7,905	10	0.818
4	Lui (2019)	College alcohol beliefs: measurement invariance, mean differences, and correlations with alcohol use outcomes across sociodemographic groups	Journal of Counseling Psychology	1	CLASS (Osberg et al., 2010)	United States	19.95	1,148	15	0.92
5	Watterson et al. (2019)	Comparing short versions of the Alcohol Use Disorders Identification Test (AUDIT) in a military cohort	BMJ Military Health	3	AUDIT (Saunders et al., 1993)	Australia	20.4	952	10	0.80
6	Zamboanga et al. (2019)	Validation of a seven-factor structure for the motives for playing drinking games measure	Assessment	1	BYAACQ (Kahler et al., 2005)	United States	22.6	1,809	23	0.89
7	Zhang et al. (2019)	Psychometric properties of a Chinese version of the Brief Young Adult Alcohol Consequences Questionnaire (B-YAACQ)	Addictive behaviors	1	BYAACQ (Kahler et al., 2005)	China	19.88	1,616	18	0.94
8	Bravo et al. (2019)*	Cross-cultural examination of negative alcohol-related consequences: measurement invariance of the young adult alcohol consequences questionnaire in Spain, Argentina, and United States	Psychological Assessment	1	CLASS (Osberg et al., 2010; Prince et al., 2018)	United States Argentina Spain	22.05 22.48 20.93	774 298 205	12	S1 0.86 S2 0.84 S3 0.87
9	Martin et al. (2020)	Optimal assessment of protective behavioral strategies among college drinkers: An item response theory analysis	Psychological Assessment	1	PDPS (Martin et al., 2020)	United States	19.99	503	20	0.95
10	Lewis et al. (2020)	Examining the ecological validity of the prototype willingness model for adolescent and young adult alcohol use	Psychology of Addictive Behaviors	1	Perceived access to alcohol and other drug scale (Kuntsche et al., 2008)	United States	18.7	124	4	0.94
11	Hallit et al. (2020)	Validation of the AUDIT scale and factors associated with alcohol use disorder in adolescents: results of a National Lebanese Study	BMC Pediatrics	1	AUDIT (Saunders et al., 1993)	Lebanon	15.42	1,810	10	0.978
12	Hawn et al. (2020)	Examination of a novel measure of trauma-related drinking to cope	Journal of Clinical Psychology	1	TRD (Hawn et al., 2020)	United States	21.9	1,896	4	0.88
13	Sánchez-García et al. (2020)	Spanish Adaptation of the Protective Behavioral Strategies Scale-20 (S-PBSS-20) and evaluation of its psychometric properties in university students	Psicothema	1	SPBSS-20 (Sánchez-García et al., 2020)	Spain	21.21	538	20	0.71
14	Tucker et al. (2020)	Utility of digital respondent driven sampling to recruit community-dwelling emerging adults for assessment of drinking and related risks	Addictive behaviors	1	BYAACQ (Kahler et al., 2005)	United States	23.64	357	24	0.90
15	Villarosa-Hurlocker et al. (2020)	Screening for alcohol use disorders in college student drinkers with the AUDIT and the USAUDIT: a receiver operating characteristic curve analysis	American Journal of Drug and Alcohol Abuse	1	AUDIT (Saunders et al., 1993)	United States	20.2	382	10	0.80
16	Delaney et al. (2020)*	The brief situational confidence questionnaire for alcohol a psychometric assessment with incarcerated youth	Psychological Assessment	1	BSCQ (Delaney et al., 2020)	United States	16.90 17.12	205 189	8	S1 0.84 S2 0.83
17	Lui et al. (2020)*	College alcohol belief and alcohol use: testing moderations by cultural orientations and ethnicity	Journal of Counseling Psychology	1	CLASS (Osberg et al., 2010)	United States	20.16 21.04	439 161	15	S1 0.91 S2 0.92
18	Motos Sellés et al. (2021)	Diagnostic utility of new short versions of AUDIT to detect binge drinking in undergraduate students	Clínica y Salud	3	AR2i (Cortés et al., 2017)	Spain	18.55	907	2	0.90
19	Rodrigues et al. (2021)	Portuguese validation of the alcohol craving questionnaire–short form–revised	PLOS ONE	1	ACQ-SF-R (Singleton et al., 1994)	Portugal	20.37	591	12	0.85
20	Davis et al. (2021)	Effects of alcohol sensitivity on alcohol-induced blackouts and passing out: An examination of the alcohol sensitivity questionnaire among underage drinkers	Alcoholism: Clinical and Experimental Research	1	ASQ (O’Neill et al., 2002)	United States	18.28	925	15	0.92
21	Jordan et al. (2021)	Psychometric validation of the protective drinking practices scale in college students across the United States	Experimental and Clinical Psychopharmacology	1	PDPS (Martin et al., 2020)	United States	22.24	684	20	0.92
22	Trager et al. (2021)*	Exploratory and confirmatory factor analysis of the parental rules toward adolescent drinking questionnaire: two factors are better than the original one	Addictive Behaviors	1	PRQ (Van der Vorst et al., 2006)	Netherlands	14.57 14.56	1,429 1,493	7	S1 0.93 S2 0.88
23	McKay et al. (2021)*	One rule for one, and a different rule for another: the case of the parental rules about alcohol questionnaire	Drug and Alcohol Dependence	1	PRQ (Van der Vorst et al., 2006)	United Kingdom	12.5 13.5 14.5 15.3 16.3	10,954 9,942 9,710 9,383 4,850	5	S1 0.88 S2 0.89 S3 0.90 S4 0.90 S5 0.87
24	Delawalla et al. (2023)	The use of MMPI-3 scales to assess personality-based vulnerabilities for alcohol use and problems	Psychological Assessment	1	AUDIT (Saunders et al., 1993)	United States	18.78	401	10	0.79
25	Gomà-i-Freixanet et al. (2023)	Assessing alcohol expectations in university students: the APNE scale	International Journal of Mental Health and Addiction	1	AUDIT (Saunders et al., 1993)	Spain	22	1,309	10	0.79
26	Stamates et al. (2023)	Validation of the brief young adult alcohol consequences questionnaire among student and nonstudent young adults	Experimental and Clinical Psychopharmacology	2	BYAACQ (Kahler et al., 2005)	United States	19.96	560	24	0.88
27	Leary et al. (2023) *	Development and validation of the Personal Assessment of Responsible Drinking Identity (PARDI) with a college student sample	Psychological Assessment	1	PARDI (Leary et al., 2023)	United States	19.48 21.59	911 1,096	20	S1 0.89 S2 0.90

Journal Quartile (Q); Indexation (SJR-Scimago Journal Reports). Studies with multiple samples (*); Sample size (S). Instruments: Alcohol Use Disorders Identification Test (AUDIT), Brief Young Adult Alcohol Consequences Questionnaire (BYAACQ), College Life Alcohol Salience Scale (CLASS), Protective Drinking Practices Scale (PDPS), Parental Rules Toward Alcohol Use (PRQ), Brief Situational Confidence Questionnaire–Alcohol (BSCQ), Alcohol Sensitivity Questionnaire (ASQ), Alcohol-Induced Blackout Measure (ABOM), Protective Behavioral Strategies Scale-20 (S-PBSS-20), Trauma-Related Drinking to Cope (TRD), Alcohol Craving Questionnaire–Short Form–Revised (ACQ-SF-R), Responsible Drinking Identity (PARDI).

### 2.1 Characteristics of the included studies

The highest frequency of publication occurred in 2020, with 10 studies representing 37.0% of the total. The journal with the highest number of publications was Psychological Assessment (*n* = 5; 18.5%). Most studies were published in Q1-indexed journals (*n* = 23; 85.2%).

In terms of geographical distribution, most studies were conducted in North America (*n* = 20; 54.1%), followed by Europe (*n* = 12; 32.4%). The United States was the country with the highest number of publications (*n* = 19; 51.4%).

The total sample across all studies comprised 78,972 participants, with a minimum sample size of 124 and a maximum of 10,954. The mean age of participants was 19.1 years (SD = 2.90), with an age range between 12.5 and 23.6 years.

The mean number of items in the psychometric instruments documented was 12.7, with a minimum of 2 and a maximum of 24 items per instrument.

### 2.2 Documented psychometric instruments

Fourteen psychometric instruments were identified (see Supplementary S4).

The most frequently used was the Alcohol Use Disorders Identification Test (AUDIT; Saunders et al., 1993), reported in 25.9% of the studies. The Brief Young Adult Alcohol Consequences Questionnaire (BYAACQ; Kahler et al., 2005) was used in 14.8%, while the College Life Alcohol Salience Scale (CLASS; Osberg et al., 2010; Prince et al., 2018) was documented in 11.3% of the studies. The Protective Drinking Practices Scale (PDPS; Martin et al., 2020) and the Parental Rules Toward Alcohol Use (PRQ; Van der Vorst et al., 2006) were each used in 7.4% of the studies.

Additional instruments identified included the AR2i (Cortés et al., 2017), Brief Situational Confidence Questionnaire–Alcohol (BSCQ; Delaney et al., 2020), Alcohol Sensitivity Questionnaire (ASQ; O’Neill et al., 2002), Alcohol-Induced Blackout Measure (ABOM; Miller et al., 2019), Perceived Access to Alcohol and Other Drug Scale (Kuntsche et al., 2008), Protective Behavioral Strategies Scale-20 (S-PBSS-20; Sánchez-García et al., 2020), Trauma-Related Drinking to Cope (TRD; Hawn et al., 2020), Alcohol Craving Questionnaire–Short Form–Revised (ACQ-SF-R; Singleton et al., 1994), and Responsible Drinking Identity (PARDI; Leary et al., 2023).

The instruments were classified based on their primary function: Assessment of risk and severity of alcohol consumption: Used in eight studies (AUDIT: Saunders et al., 1993; AR2i: Cortés et al., 2017). Evaluation of alcohol-related consequences: Used in six studies (BYAACQ: Kahler et al., 2005; ABOM: Miller et al., 2019; S-PBSS-20: Sánchez-García et al., 2020). Examination of motives and reasons for alcohol consumption: Reported in six studies (ACQ-SF-R: Singleton et al., 1994; ASQ: O’Neill et al., 2002; CLASS: Osberg et al., 2010; Prince et al., 2018; TRD: Hawn et al., 2020). Assessment of risk and protective factors: Used in seven studies (PRQ: Van der Vorst et al., 2006; Perceived Access to Alcohol and Other Drug Scale: Kuntsche et al., 2008; PDPS: Martin et al., 2020; BSCQ: Delaney et al., 2020; PARDI: Leary et al., 2023).

In the analysis of characteristics related to language, population, and application context, four instruments stand out as the most representative: The Alcohol Use Disorders Identification Test (AUDIT; Saunders et al., 1993) is the instrument with the highest number of cultural and linguistic adaptations (English, Spanish, Portuguese, Chinese, Arabic, among others). It is commonly used as a screening tool in both general and clinical populations, particularly among adults and young adults.

The Brief Young Adult Alcohol Consequences Questionnaire (BYAACQ; Kahler et al., 2005) is available in English, Spanish, and Chinese. It is frequently applied in young adult populations to identify the negative consequences associated with alcohol consumption.

The College Life Alcohol Salience Scale (CLASS; Osberg et al., 2010) was originally developed in English and later adapted into Spanish by Bravo et al. (2019). Its primary use is within university settings to assess beliefs and attitudes toward alcohol use among young adults.

The Parental Rules toward Alcohol Use Questionnaire (PRQ; van der Vorst et al., 2005; Van der Vorst et al., 2006) is available in Dutch and English. It has been employed with adolescents aged 12–18 years, particularly within parent–child dyads in the Netherlands and the United Kingdom, to examine family norms and parental rule-setting regarding alcohol use.

### 2.3 Descriptive analysis of Cronbach’s alpha and fit indices

The overall reliability (Cronbach’s alpha) across studies indicated good internal consistency among the measurement instrument items (*M* = 0.876; SD = 0.0560).

Additionally, fit indices were reported as follows: Comparative Fit Index (CFI): *M* = 0.941; SD = 0.0271 (23 studies), Tucker-Lewis Index (TLI): M = 0.918; SD = 0.0537 (14 studies), Root Mean Square Error of Approximation (RMSEA): M = 0.0655; SD = 0.0414 (23 studies), Standardized Root Mean Square Residual (SRMR): *M* = 0.0414; SD = 0.0225 (13 studies). These values suggest a good fit of the theoretical model to the observed data (Table 2).

**Table 2:** Descriptive analysis of Cronbach’s alpha and fit indices.

Statistic	Cronbach’s alpha	CFI	RMSEA	SRMR	TLI
*n*	37	23	23	13	14
Did not report	0	14	14	24	23
Mean	0.876	0.941	0.0655	0.0414	0.918
Median	0.890	0.950	0.0500	0.0380	0.921
Standard deviation	0.0550	0.0271	0.0414	0.0225	0.0537
Minimum	0.710	0.886	0.0100	0.0210	0.773
Maximum	0.978	0.994	0.155	0.104	0.982
25 percentile	0.840	0.920	0.0400	0.0238	0.906
50 percentile	0.890	0.950	0.0500	0.0380	0.921
75 percentile	0.910	0.955	0.0800	0.0500	0.952

CFI, Comparative Fit Index; RMSEA, Root Mean Square Error of Approximation; TLI, Tucker-Lewis Index; SRMR, Standardized Root Mean Square Residual.

### 2.4 Instrument-specific reliability and model-fit analysis

The most frequently used psychometric instruments- those included in two or more studies- were the Alcohol Use Disorders Identification Test (AUDIT), the Parental Rules toward Alcohol Use Questionnaire (PRQ), the College Life Alcohol Salience Scale (CLASS), and the Brief Young Adult Alcohol Consequences Questionnaire (BYAACQ) (see Supplementary S5).

Seven studies employing the AUDIT reported a mean Cronbach’s alpha of 0.83 (SD = 0.068). Model-fit indices were satisfactory: CFI averaged 0.96 (SD = 0.040; 3 studies), TLI averaged 0.97 (SD = 0.0064; 2 studies), RMSEA averaged 0.032 (SD = 0.018; 3 studies), and SRMR was 0.024 (1 study). These findings confirm strong internal consistency and adequate model fit across different cultural and population contexts.

Seven studies (from two original articles) using the PRQ showed a mean Cronbach’s alpha of 0.89 (SD = 0.020). The mean CFI was 0.94 (SD = 0.026; 7 studies), TLI averaged 0.89 (SD = 0.055; 7 studies), RMSEA averaged 0.087 (SD = 0.026; 7 studies), and SRMR averaged 0.040 (SD = 0.029; 7 studies). Although reliability values were high, some RMSEA values indicated moderate model misfit, possibly reflecting differences in sample age and item structure.

Six studies (from two original articles) applying the CLASS reported a mean Cronbach’s alpha of 0.89 (SD = 0.034). The mean CFI was 0.94 (SD = 0.014; 6 studies), TLI averaged 0.94 (SD = 0.024; 3 studies), RMSEA averaged 0.051 (SD = 0.023; 6 studies), and SRMR averaged 0.042 (SD = 0.0087; 3 studies). These results indicate good internal consistency and adequate model fit.

Four studies using the BYAACQ presented a mean Cronbach’s alpha of 0.90 (SD = 0.026). Available model-fit indices were: CFI = 0.97 (1 study), TLI = 0.91 (1 study), and RMSEA = 0.055 (1 study), while SRMR was not reported. Overall, the BYAACQ demonstrated strong reliability and satisfactory structural validity among adolescent and young adult populations.

### 2.5 Random effects model and heterogeneity statistics

The 27 studies included in this meta-analysis reported a total of 37 reliability measures. These measures were pooled, and Cronbach’s alpha reliability estimates, along with their confidence intervals, were calculated using a restricted maximum likelihood random-effects model (Table 3).

**Table 3:** Random-effects model and heterogeneity statistics.

Statistic	Cronbach’s alpha	CFI	TLI	SRMR	RMSEA
Studies	*n* = 37	*n* = 23	*n* = 14	*n* = 13	*n* = 23
Estimate	0.877	0.941	0.918	0.0375	0.0672
SE	0.00883	0.00564	0.0143	0.00860	0.0107
*Z*	99.3	167	64.1	4.35	6.28
*P*	<0.001	<0.001	<0.001	<0.001	<0.001
CI lower bound	0.859	0.930	0.890	0.021	0.046
CI upper bound	0.894	0.952	0.946	0.054	0.088
Tau	0.053	0.027	0.053	0.017	0.036
Tau^2^	0.0028 (SE = 7e-04)	7e-04 (SE = 2e-04)	0.0029 (SE = 0.0011)	3e-04 (SE = 3e-04)	0.0013 (SE = 7e-04)
I^2^	99.65%	99.89%	99.94%	35.9%	63.06%
H^2^	285.507	942.179	1713.562	1.560	2.707
Df	36	22	13	12	22
Q	9615.553	13383.862	9922.357	14.573	54.870
P	<0.001	<0.001	<0.001	<0.0266	<0.001

CFI, Comparative Fit Index; RMSEA, Root Mean Square Error of Approximation; TLI, Tucker-Lewis Index; SRMR, Standardized Root Mean Square Residual.

The reliability estimation criterion was Cronbach’s alpha > 0.70, while the fit index criteria were: Comparative Fit Index (CFI) > 0.90; Tucker-Lewis Index (TLI) > 0.90; Standardized Root Mean Square Residual (SRMR) < 0.04; Root Mean Square Error of Approximation (RMSEA) < 0.08. Heterogeneity was assessed using Cochran’s Q and the I^2^ statistic, where an I^2^ value of 50% indicates low heterogeneity. Possible publication bias was evaluated using the Funnel Plot and Egger’s test.

The pooled Cronbach’s alpha reliability coefficient was α = 0.877 (SE = 0.00883; 95% CI = 0.859–0.894). However, significant statistical heterogeneity was detected across the included studies (*I*^2^ = 99.65%; *Q* = 9615.553; *p* < 0.001), likely due to differences in sample size and instrument length.

A global sensitivity analysis of Cronbach’s alpha was conducted using four models (see Supplementary S6).

**Figure 2:**
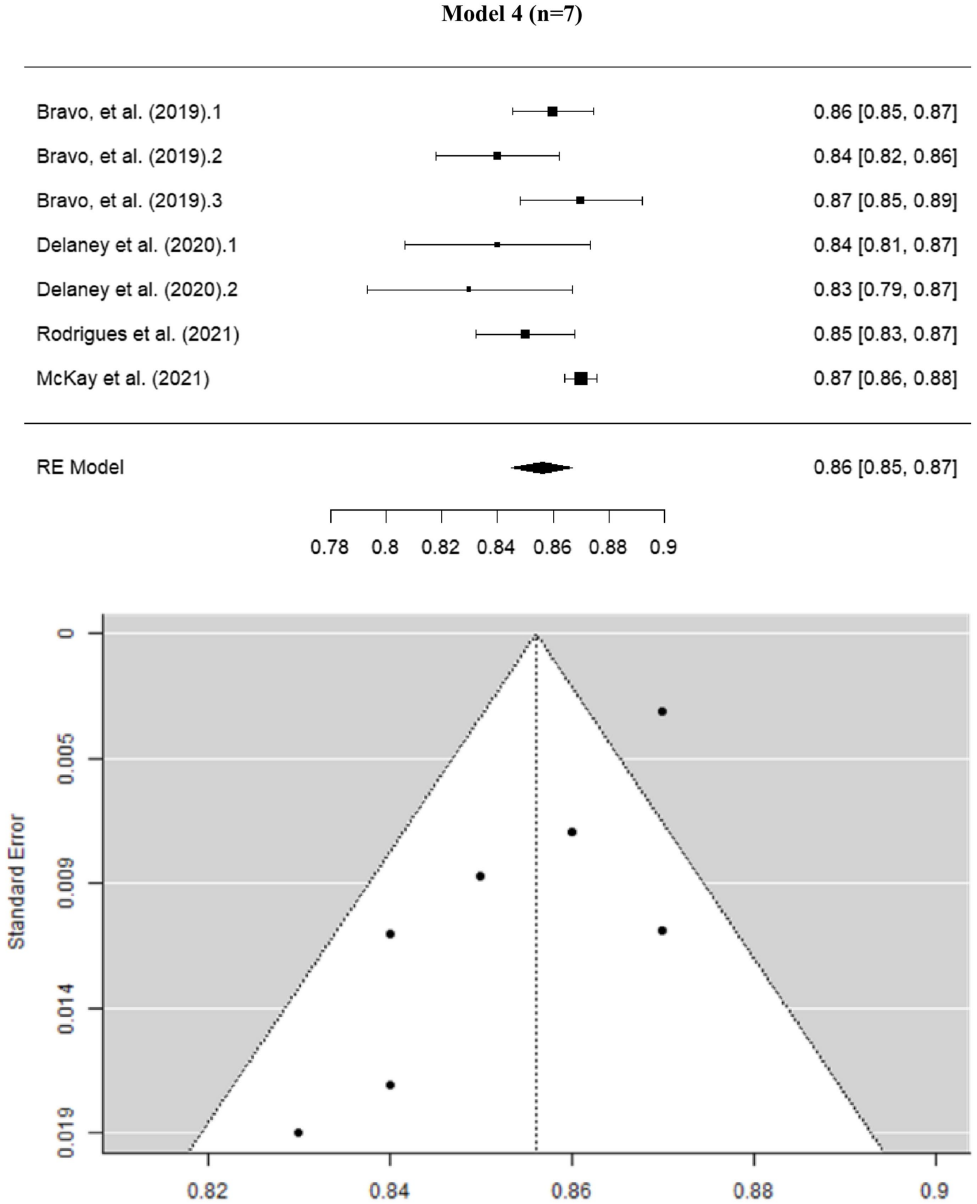
Forest and funnel plot of Cronbach’s alpha global sensitivity analysis.

The sensitivity analysis is summarized as follows: in Model 1, Egger’s coefficient was −5.301, and I^2^ was 99.65%. In the second model, studies with an alpha lower than 0.80 were removed. Minor differences were observed in the coefficients, with Egger = −3.317 and *I*^2^ = 95.5%. In the third model, studies with an alpha greater than 0.90 were excluded, resulting in coefficients of Egger = −3.109 and *I*^2^ = 98.85%. In Model 4, studies with alpha values lower than 0.83 and higher than 0.87 were removed, yielding an Egger coefficient of −3.537 and *I*^2^ = 61.73% (Figure 2).

Thus, outliers appear to influence the initial model. However, these results should be interpreted with caution. Addressing this issue will be a priority objective for future studies in the following year.

#### 2.5.1 Comparative fit index

**Figure 3:**
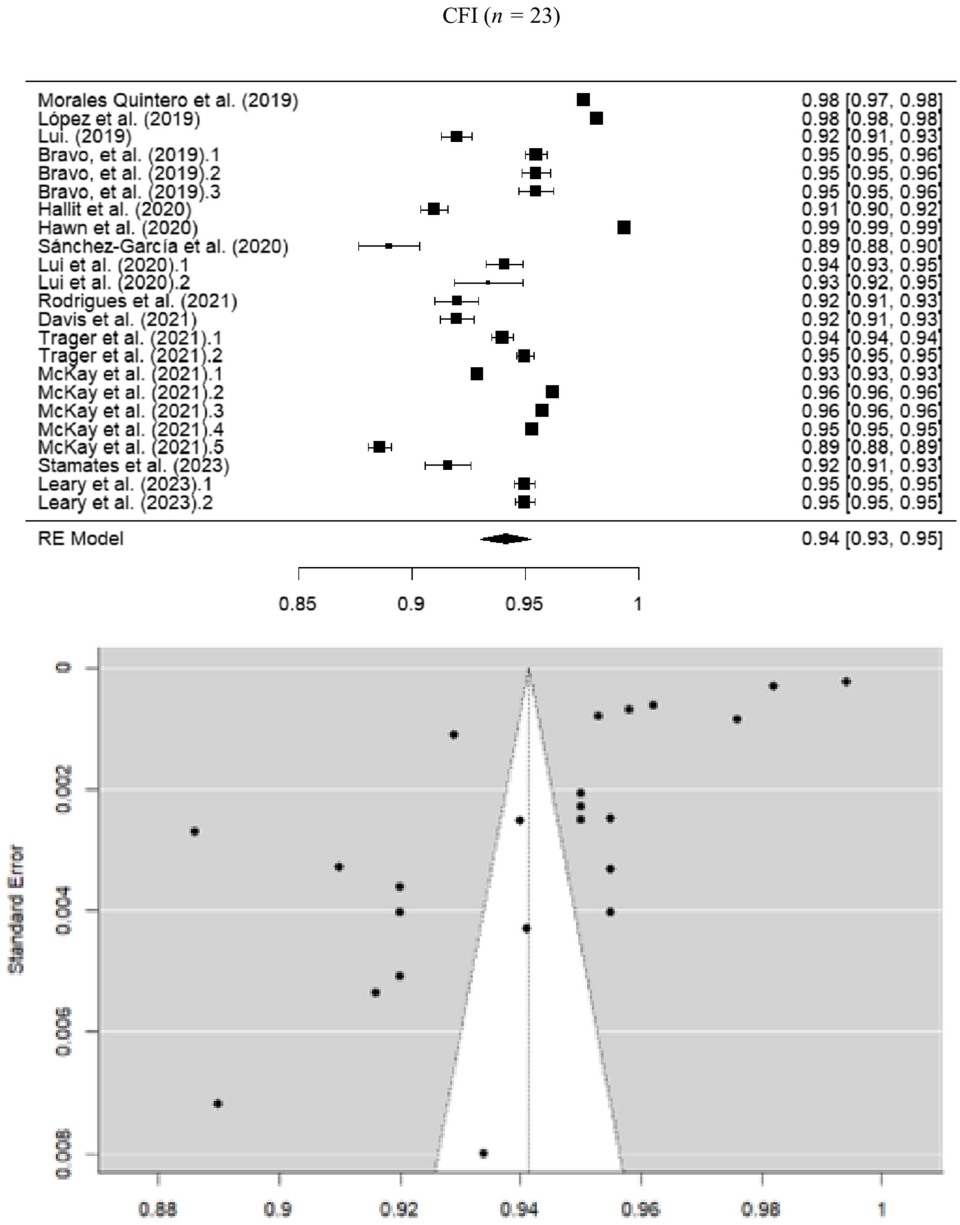
Forest and funnel plot for CFI meta-analysis.

The random-effects meta-analysis of CFI values resulted in a pooled estimate of 0.941 (SE = 0.00564; 95% CI = 0.930–0.952), indicating a good model fit. However, significant heterogeneity was observed (*I*^2^ = 99.89%; *Q* = 13383.862; *p* < 0.001), attributed to differences in sample size and instrument structure (Figure 3).

#### 2.5.2 Tucker-Lewis index

**Figure 4:**
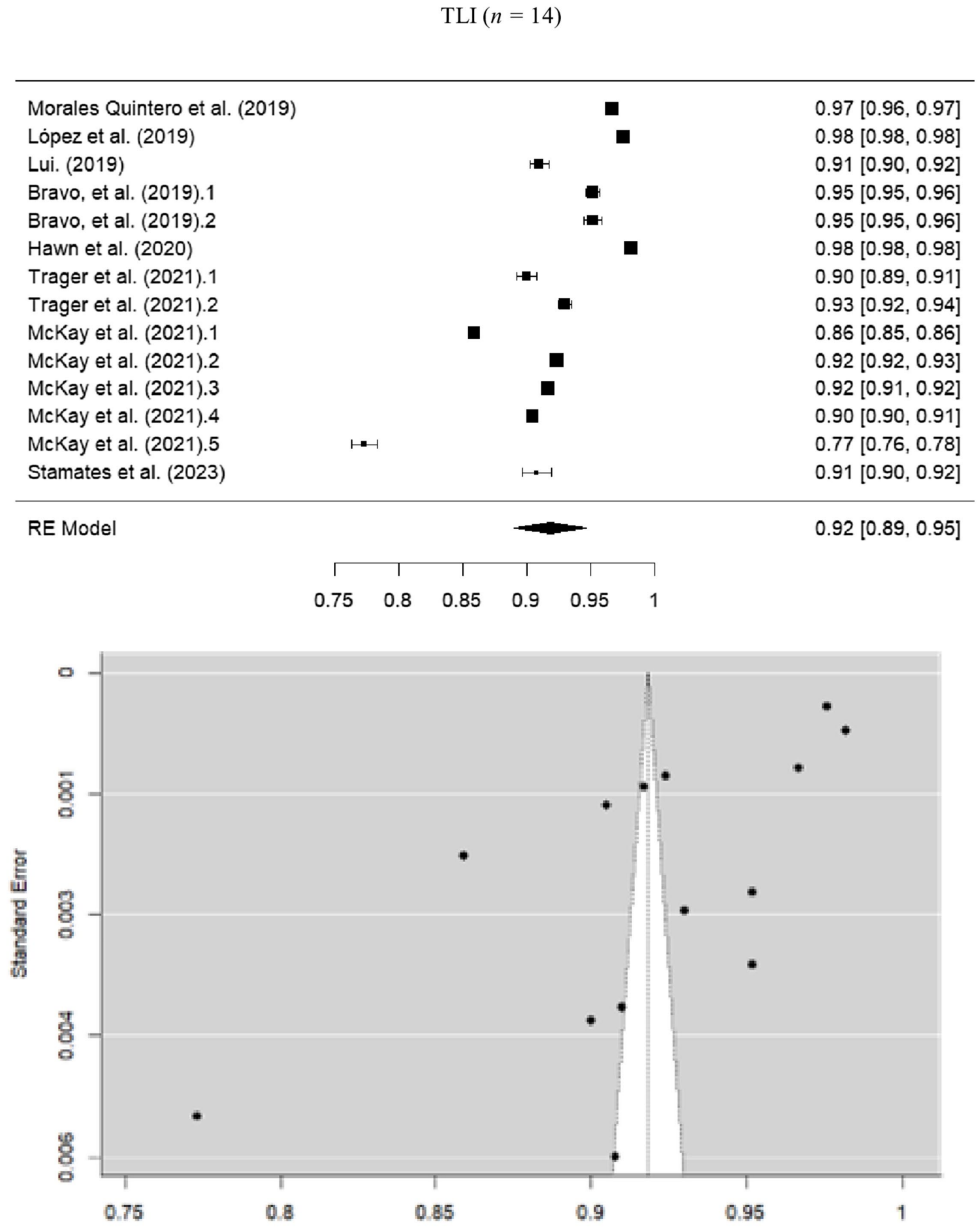
Forest and funnel plot for TLI meta-analysis.

The pooled estimate for TLI was 0.918 (SE = 0.0143; 95% CI = 0.890–0.946), suggesting a good model fit. However, high heterogeneity was detected (*I*^2^ = 99.94%; *Q* = 9922.357; *p* < 0.001), likely due to variations in sample size and number of instrument items (Figure 4).

#### 2.5.3 Root mean square error of approximation

**Figure 5:**
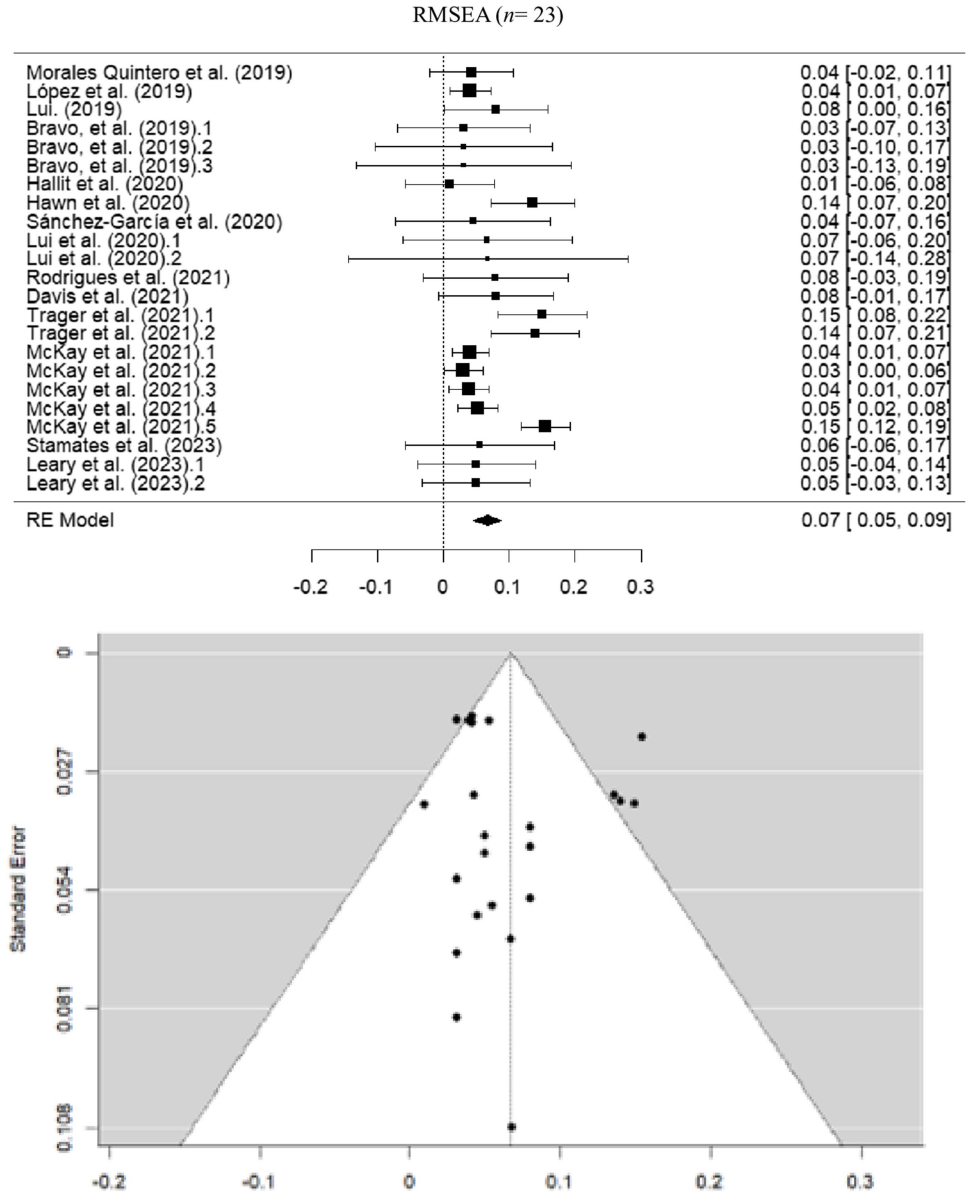
Forest and funnel plot for RMSEA meta-analysis.

The pooled estimate for RMSEA was 0.0672 (SE = 0.0107; 95% CI = 0.046–0.088), suggesting a moderately adequate fit, considering the recommended threshold of < 0.08. However, moderate heterogeneity was detected (*I*^2^ = 63.06%; *Q* = 54.870; *p* < 0.001) (Figure 5).

#### 2.5.4 Standardized root mean square residual

**Figure 6:**
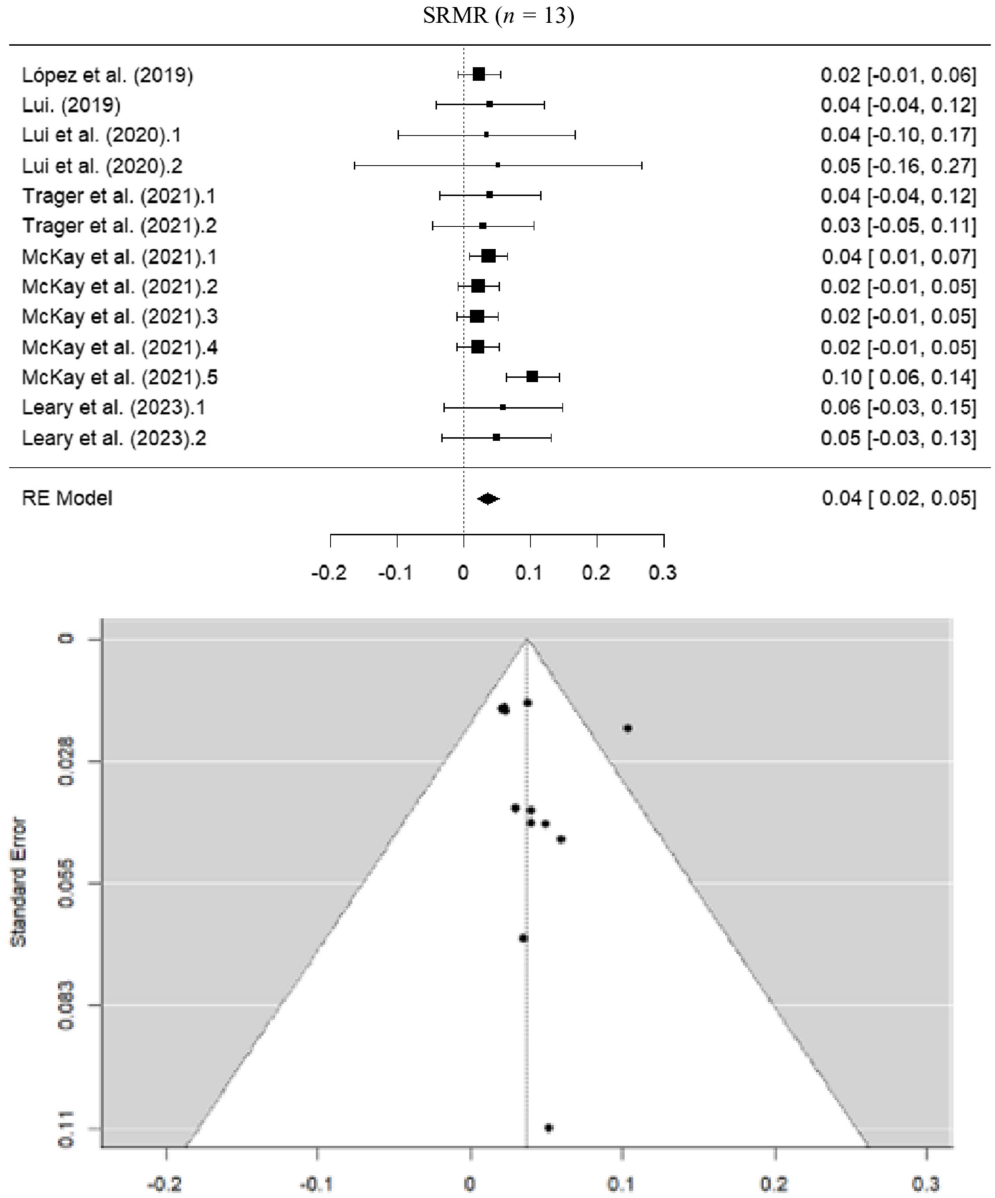
Forest and funnel plot for SRMR meta-analysis.

The pooled estimate for SRMR was 0.0375 (SE = 0.00860; 95% CI = 0.021–0.054), indicating a good model fit, given the recommended cut-off value of 0.05. The heterogeneity analysis showed moderate heterogeneity (*I*^2^ = 35.09%; *Q* = 14.573; *p* = 0.0266) (Figure 6).

## 3 Discussion

The objective of this study was to analyze the reliability of psychometric instruments used to assess alcohol consumption among adolescents and young adults. In health and social sciences, internal consistency reliability is commonly assessed using Cronbach’s alpha (Doval et al., 2023). The 27 studies included in this meta-analysis allowed us to document 27 Cronbach’s alpha measures, considering studies with multiple samples.

The Alcohol Use Disorders Identification Test (AUDIT) with a two-factor structure, was the most widely used instrument for diagnosing alcohol consumption (Morales Quintero et al., 2019; López et al., 2019; Watterson et al., 2019; Hallit et al., 2020; Villarosa-Hurlocker et al., 2020; Delawalla et al., 2023; Gomà-i-Freixanet et al., 2023). This structure includes a consumption factor with three items and a consequences factor with six items (Seay and Feely, 2020). In this meta-analysis, Cronbach’s alpha for the AUDIT ranged from 0.79 to 0.98, consistent with previous studies reporting values between 0.81 and 0.87 (Dermody et al., 2023).

The Brief Young Adult Alcohol Consequences Questionnaire (BYAACQ) was the second most frequently used instrument. This tool is particularly sensitive to detecting the negative consequences of alcohol consumption among students and demonstrates high internal consistency (Zamboanga et al., 2019; Zhang et al., 2019; Tucker et al., 2020; Stamates et al., 2023). In this meta-analysis, the BYAACQ’s Cronbach’s alpha ranged from 0.88 to 0.94.

In the comparison of the most frequently used instruments, the AUDIT shows the best psychometric balance, combining acceptable internal consistency (α = 0.83, SD = 0.067), which confirms its reliability across diverse cultural and population contexts, consistent with the findings of Toner et al. (2019) and Dermody et al. (2023). However, the present meta-analysis provides additional evidence supporting an excellent model fit (CFI = 0.96, TLI = 0.97, RMSEA = 0.032, SRMR = 0.024).

The BYAACQ exhibits the highest reliability (α = 0.90) and satisfactory model fit (CFI = 0.97, RMSEA = 0.055), although these results are based on fewer studies and with some fit indices unreported. The CLASS demonstrates good reliability (α = 0.89) and acceptable fit (CFI = 0.94, RMSEA = 0.051), particularly in university samples; however, not all fit indices were reported. In contrast, the PRQ shows strong internal consistency (α = 0.89) but a higher RMSEA value (0.087) and incomplete reporting of fit indices, suggesting moderate structural misfit.

### 3.1 Reliability

The 37 reliability measures analyzed in this meta-analysis indicate good internal consistency across the psychometric instruments. The overall Cronbach’s alpha estimate was 0.88 (95% CI: 0.86–0.89), aligning with the recommended range of 0.80 to 0.90 (Streiner, 2003; Oviedo and Campo-Arias, 2005). All instruments exceeded the 0.70 threshold despite being applied in diverse cultural contexts, including Mexico, Ecuador, Argentina, Spain, Portugal, the United States, the United Kingdom, the Netherlands, Australia, China, and Lebanon.

Based on the interpretation guidelines by George and Mallery (2010), no instrument in this study reported a questionable Cronbach’s alpha (below 0.70). Instead: Three instruments had acceptable Cronbach’s alpha values above 0.70 (Sánchez-García et al., 2020; Delawalla et al., 2023; Gomà-i-Freixanet et al., 2023). Eighteen instruments reported Cronbach’s alpha above 0.80 (Morales Quintero et al., 2019; López et al., 2019; Watterson et al., 2019; Zamboanga et al., 2019; Hawn et al., 2020; Villarosa-Hurlocker et al., 2020; Bravo et al., 2019; Delaney et al., 2020; Rodrigues et al., 2021; Trager et al., 2021; McKay et al., 2021; Stamates et al., 2023; Leary et al., 2023). Sixteen instruments showed excellent Cronbach’s alpha values exceeding 0.90 (Zhang et al., 2019; Miller et al., 2019; Lui, 2019; Tucker et al., 2020; Martin et al., 2020; Lewis et al., 2020; Hallit et al., 2020; Lui et al., 2020; Trager et al., 2021; Motos Sellés et al., 2021; Davis et al., 2021; McKay et al., 2021; Jordan et al., 2021; Leary et al., 2023).

However, very high Cronbach’s alpha values may indicate item redundancy or overfitting, leading to overestimated internal consistency (Streiner, 2003). For example, an alpha of 0.98 (Hallit et al., 2020) suggests potential item duplication. The optimal threshold for Cronbach’s alpha is recommended at 0.90 ± 0.02, with values above this suggesting redundancy (Streiner, 2003).

In the context of this meta-analysis, high alpha values may inflate pooled reliability estimates and increase heterogeneity across studies. This effect may result from item redundancy rather than genuine internal consistency, which helps to interpret the aggregated reliability more accurately. This finding also underscores the need to complement alpha with additional reliability indicators, such as McDonald’s omega (*ω*), which is based on factor analysis and provides a more comprehensive evaluation of the internal structure (Hayes and Coutts, 2020).

### 3.2 Psychometric fit indices

The Comparative Fit Index (CFI) = 0.94 and Tucker-Lewis Index (TLI) = 0.92 indicate good model fit. However, a high degree of heterogeneity among studies was observed, likely due to differences in sample size, number of items, and sociocultural characteristics, rather than random variation. The Root Mean Square Error of Approximation (RMSEA) = 0.067 suggests a moderate fit, aligning with Hoogland and Boomsma’s (1998) recommendation that values below 0.05 are considered optimal. Similarly, the Standardized Root Mean Square Residual (SRMR) = 0.040 indicates an acceptable fit for the meta-analysis model. Heterogeneity was moderate for RMSEA and low for SRMR.

Variability in the models reflects real differences between studies rather than random error. This underscores the importance of considering heterogeneity when interpreting meta-analysis results, particularly for clinical applications or future research. Fit indices should always be evaluated collectively and within the study’s specific context.

### 3.3 Limitations

One limitation of this meta-analysis is its reliance on Cronbach’s alpha as the primary reliability metric. While widely used, Cronbach’s alpha assumes unidimensionality, tau-equivalence (equal factor loadings), and independent errors, which may not always hold true (Doval et al., 2023; Kalkbrenner, 2024). The indiscriminate use of Cronbach’s alpha has led to its misconception as the “gold standard” of reliability, despite its limitations (Elosua Oliden and Zumbo, 2008). Cronbach’s alpha depends on several statistical assumptions, including normality of test scores, unidimensionality, unit-weighted scores, essential tau-equivalence, and independent errors (Edwards et al., 2021). It is more informative when complemented with McDonald’s Omega (ω), which provides a more robust measure of reliability (Cho and Kim, 2015; Trizano-Hermosilla and Alvarado, 2016).

To strengthen future meta-analyses, studies should report additional psychometric fit indices Comparative Fit Index (CFI), Tucker-Lewis Index (TLI), Root Mean Square Error of Approximation (RMSEA), Standardized Root Mean Square Residual (SRMR) and Average Variance Extracted (AVE), which helps evaluate construct validity. Not all studies report fit indices, limiting meta-analytic insights into the reliability of psychometric tools.

Second, heterogeneity analysis is essential in meta-analysis (Higgins and Thompson, 2002). Bias in reliability estimation may stem from differences in sample characteristics, psychometric tools, or sociodemographic factors. To enhance accuracy, sensitivity analyses should sequentially exclude studies to determine their impact on heterogeneity (Patsopoulos et al., 2008).

Finally, cultural and demographic variations—across North America, South America, Europe, Asia, and Oceania—must be considered when interpreting results. Differences in sample size, instrument structure, and sociocultural contexts may introduce variability in psychometric estimates. As Cortés et al. (2021) emphasizes, these contextual aspects can influence the validity and reliability of measurement instruments and should therefore be carefully examined in future cross-cultural research.

### 3.4 Strengths

First, it integrates two of the most widely recognized methodological standards for psychometric evaluation: the Quality Criteria for Measurement Properties proposed by Terwee et al. (2007) and the COSMIN Guidelines for systematic reviews of outcome measurement instruments (Mokkink et al., 2024). The joint application of these frameworks ensures a transparent and reproducible process for assessing measurement quality and minimizes risk of bias in the synthesis of psychometric evidence.

Second, the meta-analysis applies a restricted maximum likelihood random-effects model, which strengthens the precision and generalizability of pooled reliability estimates. Third, the inclusion and evaluation of multiple model fit indices (CFI, TLI, RMSEA, SRMR) expand the evidence base beyond internal consistency, offering a comprehensive perspective on structural and construct validity across diverse samples.

Finally, although cultural variability was identified as a potential source of heterogeneity, the broad international coverage of the included studies -spanning North America, South America, Europe, Asia, and Oceania- also represents a major strength. This diversity enhances the external validity of the findings and underscores the cross-cultural relevance of psychometric research on alcohol-related instruments.

### 3.5 Conclusion

This systematic review and meta-analysis demonstrate that all included studies reported Cronbach’s alpha values above 0.70, with an overall mean Cronbach’s alpha of 0.88. The psychometric properties of the instruments used to assess alcohol consumption among adolescents and young adults are adequate and reliable.

The Alcohol Use Disorders Identification Test (AUDIT) was the most frequently used and demonstrated strong reliability (α > 0.70 in all cases). The Brief Young Adult Alcohol Consequences Questionnaire (BYAACQ) also exhibited high internal consistency (α > 0.80 in all studies). Together, these two instruments emerge as the most psychometrically robust options for assessing alcohol-related behaviors in this population.

According to the criteria (Terwee and COSMIN), most instruments achieved a “positive” or “adequate” rating across key domains of internal consistency, structural validity, and content validity. The AUDIT and BYAACQ demonstrated the highest overall methodological quality and lowest risk of bias. Instruments such as CLASS, PRQ, and ACQ-SF-R were rated as “intermediate” mainly due to incomplete reporting of model fit indices or limited cross-cultural validation evidence.

From a practical and clinical perspective, the AUDIT and BYAACQ stand out as reliable, cost-effective, and easy-to-administer tools for detecting alcohol risk patterns and consequences in adolescents and young adults. Their psychometric robustness supports their application not only in research but also in school-based screenings, primary care settings, and community prevention programs, where early detection of risky consumption can improve timely intervention and reduce long-term harm.

The incorporation of standardized methodological frameworks, such as the Terwee criteria and COSMIN checklist, strengthens the interpretive validity of these conclusions and minimizes potential bias in synthesizing psychometric evidence. This approach establishes a benchmark for future studies aiming to evaluate and compare alcohol-related measurement tools using reproducible and transparent quality standards.

Finally, integrating these tools into clinical practice and public health initiatives can enhance the identification of hazardous drinking behaviors, promote individualized interventions, and improve the monitoring of preventive and therapeutic outcomes. Future research should prioritize cross-cultural adaptation and longitudinal validation of these instruments, in alignment with COSMIN guidelines, to ensure their reliability, validity, and responsiveness across diverse sociocultural contexts.

## Data Availability

The original contributions presented in the study are included in the article/Supplementary material, further inquiries can be directed to the corresponding author.

## References

[ref1] Bravo A. J. Pilatti A. Pearson M. R. Read J. P. Mezquita L. Ibáñez M. I. . (2019). Cross-cultural examination of negative alcohol-related consequences: measurement invariance of the young adult alcohol consequences questionnaire in Spain, Argentina, and USA. Psychol. Assess. 31, 631–642. doi: 10.1037/pas0000689, 30667265 PMC6488382

[ref2] Callinan S. Livingston M. Dietze P. Gmel G. Room R. (2022). Age-based differences in quantity and frequency of consumption when screening for harmful alcohol use. Addiction 117, 2431–2437. doi: 10.1111/add.15904, 35466478 PMC9544839

[ref3] Campbell K. W. Pebley K. MacKillop J. Murphy J. G. (2022). Measurement invariance of the young adult alcohol consequences questionnaire across college status, race, and childhood SES in a diverse community sample. Psychol. Addict. Behav. 36, 824–836. doi: 10.1037/adb0000789, 34647776 PMC9008066

[ref4] Cho E. Kim S. (2015). Cronbach’s coefficient alpha: well known but poorly understood. Organ. Res. Methods 18, 207–230. doi: 10.1177/1094428114555994

[ref6] Cortés M. L. Morales-Quintero L. A. Rojas J. L. Moral M. V. Flores M. Rodríguez-Díaz F. J. (2021). Alcohol consumption patterns and perception of risk in Mexican students. Rev. Iberoam. Psicol. Salud 12, 17–33. doi: 10.23923/j.rips.2021.01.042

[ref5] Cortés M. T. Giménez-Costa J. A. Motos-Sellés P. Sancerni-Beitia M. D. (2017). Revision of AUDIT consumption items to improve the screening of youth binge drinking. Front. Psychol. 8:910. doi: 10.3389/fpsyg.2017.00910, 28642722 PMC5463274

[ref7] Cox M. Chaney B. McDonald L. Beth Miller M. (2022). Assessing alcohol use in situ: correlates of self-report vs. objective alcohol consumption. Addict. Behav. 129:107278. doi: 10.1016/j.addbeh.2022.107278, 35217414 PMC9347371

[ref8] Davis C. N. Piasecki T. M. Bartholow B. D. Slutske W. S. (2021). Effects of alcohol sensitivity on alcohol-induced blackouts and passing out: an examination of the alcohol sensitivity questionnaire among underage drinkers. Alcohol. Clin. Exp. Res. 45, 1149–1160. doi: 10.1111/acer.14607, 33755998 PMC8131246

[ref9] Delaney D. J. Bernstein M. H. Harlow L. L. Farrow M. Martin R. A. Stein L. A. R. (2020). The brief situational confidence questionnaire for alcohol: a psychometric assessment with incarcerated youth. Psychol. Assess. 32, 254–264. doi: 10.1037/pas0000780, 31697110

[ref10] Delawalla C. N. Lee T. T. C. Keen M. A. (2023). The use of MMPI-3 scales to assess personality-based vulnerabilities for alcohol use and problems. Psychol. Assess. 35, 633–645. doi: 10.1037/pas0001245, 37261757

[ref11] Dermody S. S. Uhrig A. Moore A. Raessi T. Abramovich A. (2023). A narrative systematic review of the gender inclusivity of measures of harmful drinking and their psychometric properties among transgender adults. Addiction 118, 1649–1660. doi: 10.1111/add.16212, 37070479

[ref12] Doval E. Viladrich C. Angulo-Brunet A. (2023). Coefficient alpha: the resistance of a classic. Psicothema 1, 5–20. doi: 10.7334/psicothema2022.321, 36695846

[ref13] Duffy F. F. Sudom K. Jones M. Fear N. T. Greenberg N. Adler A. M. . (2023). Calibrating the alcohol use disorders identification test-consumption (AUDIT-C) for detecting alcohol-related problems among Canadian, UK and US soldiers: cross-sectional pre-deployment and post-deployment survey results. BMJ Open 13:e068619. doi: 10.1136/bmjopen-2022-068619, 37130676 PMC10163557

[ref14] Edwards A. A. Joyner K. J. Schatschneider C. (2021). A simulation study on the performance of different reliability estimation methods. Educ. Psychol. Meas. 81, 1089–1117. doi: 10.1177/0013164421994184, 34565817 PMC8451020

[ref15] Elosua Oliden P. Zumbo B. D. (2008). Coeficientes de fiabilidad para escalas de respuesta categórica ordenada. Psicothema 20, 896–901. 18940100

[ref16] George D. Mallery P. (2010). SPSS for windows step by step: a simple study guide and reference, 17.0 update, 10/e. Boston: Pearson Education India.

[ref17] Gomà-i-Freixanet M. Ferrero-Rincón G. Granero R. (2023). Assessing alcohol expectations in university students: the APNE scale. Int. J. Ment. Health Addict. 21, 4259–4274. doi: 10.1007/s11469-022-00854-6

[ref18] Hadland S. E. Knight J. R. Harris S. K. (2019). Alcohol use disorder: a pediatric-onset condition needing early detection and intervention. Pediatrics 143:e20183654. doi: 10.1542/peds.2018-3654, 30783023 PMC6398366

[ref19] Hakstian A. R. Whalen T. E. (1976). A K-sample significance test for independent alpha coefficients. Psychometrika 41, 219–231. doi: 10.1007/BF02291840

[ref20] Hallit J. Salameh P. Haddad C. Sacre H. Soufia M. Akel M. . (2020). Validation of the AUDIT scale and factors associated with alcohol use disorder in adolescents: results of a National Lebanese Study. BMC Pediatr. 20:205. doi: 10.1186/s12887-020-02116-7, 32393212 PMC7212566

[ref21] Harrer M. Cuijpers P. Furukawa T. Ebert D. (2021). Doing meta-analysis with R: A hands-on guide: Chapman and Hall/CRC. doi: 10.1201/9781003107347

[ref22] Hawn S. E. Aggen S. H. Cusack S. E. Spit for Science Working Group Dick D. Amstadter A. B. (2020). Examination of a novel measure of trauma-related drinking to cope. J. Clin. Psychol. 76, 1938–1964. doi: 10.1002/jclp.22972, 32478444 PMC7721863

[ref23] Hayes A. F. Coutts J. J. (2020). Use omega rather than Cronbach’s alpha for estimating reliability. But…. Commun. Methods Meas. 14, 1–24. doi: 10.1080/19312458.2020.1718629

[ref24] Higgins J. P. Thompson S. G. (2002). Quantifying heterogeneity in a meta-analysis. Stat. Med. 21, 1539–1558. doi: 10.1002/sim.1186, 12111919

[ref25] Hoogland J. J. Boomsma A. (1998). Robustness studies in covariance structure modeling: an overview and a meta-analysis. Sociol. Methods Res. 26, 329–367. doi: 10.1177/0049124198026003003

[ref26] Hummer J. F. Tucker J. S. Rodriguez A. Davis J. P. D'Amico E. J. (2022). A longitudinal study of alcohol and Cannabis use in young adulthood: exploring racial and ethnic differences in the effects of peer and parental influences from middle adolescence. J. Stud. Alcohol Drugs 83, 684–694. doi: 10.15288/jsad.21-00050, 36136439 PMC9523756

[ref27] Jordan H. R. Colvin K. F. Kim K. Y. E. Martin J. L. Madson M. B. (2021). Psychometric validation of the protective drinking practices scale in college students across the United States. Exp. Clin. Psychopharmacol. 29, 251–260. doi: 10.1037/pha0000471, 34264736

[ref28] Kahler C. W. Strong D. R. Read J. P. (2005). Toward efficient and comprehensive measurement of the alcohol problems continuum in college students: the brief young adult alcohol consequences questionnaire. Alcoholism 29, 1180–1189. doi: 10.1097/01.alc.0000171940.95813.a, 16046873

[ref29] Kalkbrenner M. T. (2024). Choosing between Cronbach’s coefficient alpha, McDonald’s coefficient omega, and coefficient H: confidence intervals and the advantages and drawbacks of interpretive guidelines. Meas. Eval. Couns. Dev. 57, 93–105. doi: 10.1080/07481756.2023.2283637

[ref30] Klimkiewicz A. Jakubczyk A. Mach A. Abramowska M. Szczypiński J. Berent D. . (2021). Psychometric properties of the polish version of the alcohol use disorders identification test (AUDIT). Drug Alcohol Depend. 218:108427. doi: 10.1016/j.drugalcdep.2020.108427, 33250385

[ref31] Kuntsche E. Kuendig H. Gmel G. (2008). Alcohol outlet density, perceived availability and adolescent alcohol use: a multilevel structural equation model. J. Epidemiol. Community Health 62, 811–816. doi: 10.1136/jech.2007.065367, 18701732

[ref32] Leary A. V. Dvorak R. D. Burr E. K. Peterson R. De Leon A. N. Klaver S. J. . (2023). Development and validation of the personal assessment of responsible drinking identity (PARDI) with a college student sample. Psychol. Assess. 35, 618–632. doi: 10.1037/pas0001236, 37227839 PMC10384221

[ref33] Lewis M. A. Litt D. M. King K. M. Fairlie A. M. Waldron K. A. Garcia T. A. . (2020). Examining the ecological validity of the prototype willingness model for adolescent and young adult alcohol use. Psychol. Addict. Behav. 34, 293–302. doi: 10.1037/adb0000533, 31750697 PMC7064386

[ref34] López V. Paladines B. Vaca S. Cacho R. Fernández-Montalvo J. Ruisoto P. (2019). Psychometric properties and factor structure of an Ecuadorian version of the alcohol use disorders identification test (AUDIT) in college students. PLoS One 14:e0219618. doi: 10.1371/journal.pone.0219618, 31291363 PMC6619822

[ref35] Lui P. P. (2019). College alcohol beliefs: measurement invariance, mean differences, and correlations with alcohol use outcomes across sociodemographic groups. J. Couns. Psychol. 66, 487–495. doi: 10.1037/cou0000338, 30777775

[ref36] Lui P. P. Berkley S. R. Zamboanga B. L. (2020). College alcohol belief and alcohol use: testing moderations by cultural orientations and ethnicity. J. Couns. Psychol. 67, 184–194. doi: 10.1037/cou0000374, 31343217

[ref37] Martin J. L. Colvin K. F. Madson M. B. Zamboanga B. L. Pazienza R. (2020). Optimal assessment of protective behavioral strategies among college drinkers: an item response theory analysis. Psychol. Assess. 32, 394–406. doi: 10.1037/pas0000799, 31999144 PMC7163160

[ref38] McKay M. T. Perry J. L. Cole J. C. Percy A. Sumnall H. R. (2021). One rule for one, and a different rule for another: the case of the parental rules about alcohol questionnaire. Drug Alcohol Depend. 225:108824. doi: 10.1016/j.drugalcdep.2021.108824, 34186445

[ref39] Miller M. B. DiBello A. M. Merrill J. E. Carey K. B. (2019). Development and initial validation of the alcohol-induced blackout measure. Addict. Behav. 99:106079. doi: 10.1016/j.addbeh.2019.106079, 31442787 PMC6791777

[ref40] Mokkink L. B. Elsman E. B. M. Terwee C. B. (2024). The COSMIN guideline for systematic reviews of patient-reported outcome measures (PROMs). Qual. Life Res. 33, 2929–2939. doi: 10.1007/s11136-024-03761-6, 39198348 PMC11541334

[ref41] Morales Quintero L. A. Moral Jiménez M. V. Rojas Solís J. L. Bringas Molleda C. Soto Chilaca A. Rodríguez Díaz F. J. (2019). Psychometric properties of the alcohol use disorders identification test (AUDIT) in adolescents and young adults from southern Mexico. Alcohol 81, 39–46. doi: 10.1016/j.alcohol.2019.05.002, 31199963

[ref42] Motos Sellés P. Cortés Tomás M. T. Giménez Costa J. A. (2021). Diagnostic utility of new short versions of AUDIT to detect binge drinking in undergraduate students. Clín. Salud 32, 49–54. doi: 10.5093/clysa2020a23

[ref43] O’Neill S. E. Sher K. J. Bartholow B. D. (2002). Alcohol susceptibility and tolerance in young adults. Alcoholism 26:119A.

[ref44] O'Brien H. Callinan S. Livingston M. Doyle J. S. Dietze P. M. (2020). Population patterns in alcohol use disorders identification test (AUDIT) scores in the Australian population; 2007-2016. Aust. N. Z. J. Public Health 44, 462–467. doi: 10.1111/1753-6405.13043, 33104260

[ref45] Ohtani Y. Ueno F. Kimura M. Matsushita S. Mimura M. Uchida H. (2023). Highly endorsed screening and assessment scales for alcohol problems: a systematic review. Neuropsychopharmacol. Rep. 43, 470–481. doi: 10.1002/npr2.12363, 37392159 PMC10739151

[ref46] Osberg T. M. Atkins L. Buchholz L. Shirshova V. Swiantek A. Whitley J. . (2010). Development and validation of the college life alcohol salience scale: a measure of beliefs about the role of alcohol in college life. Psychol. Addict. Behav. 24, 1–12. doi: 10.1037/a0018197, 20307107

[ref47] Ouzzani M. Hammady H. Fedorowicz Z. Elmagarmid A. (2016). Rayyan-a web and mobile app for systematic reviews. Syst. Rev. 5:210. doi: 10.1186/s13643-016-0384-4, 27919275 PMC5139140

[ref48] Oviedo H. C. Campo-Arias A. (2005). Aproximación al uso del coeficiente alfa de Cronbach. Rev. Colomb. Psiquiatr. 34, 572–580.

[ref49] Page M. J. McKenzie J. E. Bossuyt P. M. Boutron I. Hoffmann T. C. Mulrow C. D. . (2021). The PRISMA 2020 statement: an updated guideline for reporting systematic reviews. BMJ 372:n71. doi: 10.1136/bmj.n71, 33782057 PMC8005924

[ref50] Patsopoulos N. A. Evangelou E. Ioannidis J. P. (2008). Sensitivity of between-study heterogeneity in meta-analysis: proposed metrics and empirical evaluation. Int. J. Epidemiol. 37, 1148–1157. doi: 10.1093/ije/dyn065, 18424475 PMC6281381

[ref51] Prince M. A. Pearson M. R. Bravo A. J. Montes K. S. (2018). A quantification of the alcohol use-consequences association in college student and clinical populations: a large, multi-sample study. Am. J. Addict. 27, 116–123. doi: 10.1111/ajad.12686, 29356194 PMC5831488

[ref52] Ramírez A. Burgos-Benavides L. Sinchi H. Quito-Calle J. V. Herrero Díez F. Rodríguez-Díaz F. J. (2025). Adaptation and validation of psychological assessment questionnaires using confirmatory factor analysis: a tutorial for planning and reporting analysis. Preprints. doi: 10.20944/preprints202502.1192.v1

[ref53] Rodrigues R. López-Caneda E. Almeida-Antunes N. Sampaio A. Crego A. (2021). Portuguese validation of the alcohol craving questionnaire-short form-revised. PLoS One 16:e0251733. doi: 10.1371/journal.pone.0251733, 34029320 PMC8143387

[ref54] Sánchez-García M. Lozano-Rojas Ó. M. Díaz-Batanero C. Carmona-Márquez J. Rojas-Tejada A. J. Fernández-Calderón F. (2020). Spanish adaptation of the protective behavioral strategies Scale-20 (S-PBSS-20) and evaluation of its psychometric properties in university students. Psicothema 32, 598–606. doi: 10.7334/psicothema2020.172, 33073767

[ref55] Saunders J. B. Aasland O. G. Babor T. F. de la Fuente J. R. Grant M. (1993). Development of the alcohol use disorders identification test (AUDIT): WHO collaborative project on early detection of persons with harmful alcohol consumption--II. Addiction 88, 791–804. doi: 10.1111/j.1360-0443.1993.tb02093.x, 8329970

[ref56] Seay K. D. Feely M. (2020). Assessment of the validity of the AUDIT factor structure in parents involved with child protective services. Am. J. Drug Alcohol Abuse 46, 546–552. doi: 10.1080/00952990.2020.1722685, 32134690 PMC8103549

[ref9002] Singleton E. G. Henningfield J. E. Tiffany S. (1994). Alcohol craving questionnaire: ACQ-Now: background and administration manual. Baltimore: NIDA Addiction Research Centre.

[ref58] Smit K. Voogt C. Otten R. Kleinjan M. Kuntsche E. (2020). Alcohol expectancies change in early to middle adolescence as a function of the exposure to parental alcohol use. Drug Alcohol Depend. 211:107938. doi: 10.1016/j.drugalcdep.2020.107938, 32222262

[ref59] Stamates A. L. Yang M. Lau-Barraco C. (2023). Validation of the brief young adult alcohol consequences questionnaire among student and nonstudent young adults. Exp. Clin. Psychopharmacol. 31, 643–651. doi: 10.1037/pha0000615, 36355679 PMC10249775

[ref60] Stephenson M. Riitano D. Wilson S. Leonardi-Bee J. Mabire C. Cooper K. . (2020). “Chapter 12: systematic reviews of measurement properties” in JBI manual for evidence synthesis. eds. Aromataris E. Munn Z. (Adelaida, Australia: JBI).

[ref61] Streiner D. L. (2003). Starting at the beginning: an introduction to coefficient alpha and internal consistency. J. Pers. Assess. 80, 99–103. doi: 10.1207/S15327752JPA8001_18, 12584072

[ref62] Terwee C. B. Bot S. D. de Boer M. R. van der Windt D. A. Knol D. L. Dekker J. . (2007). Quality criteria were proposed for measurement properties of health status questionnaires. J. Clin. Epidemiol. 60, 34–42. doi: 10.1016/j.jclinepi.2006.03.012, 17161752

[ref63] The Jamovi Project (2023). jamovi. (Version 2.4) [Computer Software]. Available online at: https://www.jamovi.org (Accessed April 07, 2024).

[ref64] Toner P. Böhnke J. R. Andersen P. McCambridge J. (2019). Alcohol screening and assessment measures for young people: a systematic review and meta-analysis of validation studies. Drug Alcohol Depend. 202, 39–49. doi: 10.1016/j.drugalcdep.2019.01.030, 31299552

[ref65] Trager B. M. Koning I. M. Turrisi R. (2021). Exploratory and confirmatory factor analysis of the parental rules toward adolescent drinking questionnaire: two factors are better than the original one. Addict. Behav. 117:106855. doi: 10.1016/j.addbeh.2021.106855, 33621921 PMC8582330

[ref66] Trizano-Hermosilla I. Alvarado J. M. (2016). Best alternatives to Cronbach's alpha reliability in realistic conditions: congeneric and asymmetrical measurements. Front. Psychol. 7:769. doi: 10.3389/fpsyg.2016.00769, 27303333 PMC4880791

[ref67] Tucker J. A. Bacon J. P. Chandler S. D. Lindstrom K. Cheong J. (2020). Utility of digital respondent driven sampling to recruit community-dwelling emerging adults for assessment of drinking and related risks. Addict. Behav. 110:106536. doi: 10.1016/j.addbeh.2020.106536, 32711287 PMC7329684

[ref68] Van der Vorst H. Engels R. C. Meeus W. Deković M. (2006). The impact of alcohol-specific rules, parental norms about early drinking and parental alcohol use on adolescents drinking behavior. J. Child Psychol. Psychiatry 47, 1299–1306. doi: 10.1111/j.1469-7610.2006.01680.x, 17176385

[ref69] Villarosa-Hurlocker M. C. Schutts J. W. Madson M. B. Jordan H. R. Whitley R. B. Mohn R. C. (2020). Screening for alcohol use disorders in college student drinkers with the AUDIT and the USAUDIT: a receiver operating characteristic curve analysis. Am. J. Drug Alcohol Abuse 46, 531–545. doi: 10.1080/00952990.2020.1712410, 32175778 PMC7492430

[ref9001] Van der Vorst H. Engels R. C. Meeus W. Deković M. Van Leeuwe J. (2005). The role of alcohol-specific socialization in adolescents’ drinking behaviour. Addiction (Abingdon, England), 100, 1464–1476. doi: 10.1111/j.1360-0443.2005.01193.x 16185208

[ref70] Voss A. T. Floyd R. G. Campbell K. W. Dennhardt A. A. MacKillop J. Murphy J. G. (2021). Psychometric evaluation of the reward probability index in emerging adult drinkers. Psychol. Addict. Behav. 35, 432–443. doi: 10.1037/adb0000712, 33764088 PMC8184582

[ref71] Watterson J. Gabbe B. Dietze P. Bowring A. Rosenfeld J. V. (2019). Comparing short versions of the alcohol use disorders identification test (AUDIT) in a military cohor. J. R. Army Med. Corps 165, 312–316. doi: 10.1136/jramc-2018-001024, 30341169

[ref72] World Health Organization 2021 Global alcohol action plan 2022–2030 to strengthen implementation of the Global Strategy to Reduce the Harmful Use of Alcohol. Available online at: https://www.who.int/publications/m/item/global-alcohol-action-plan-second-draft-unedited (Accessed June 10, 2024).

[ref73] World Health Organization 2023 World health statistics: monitoring health for the SDGs, Sustainable Development Goals. Available online at: https://www.who.int/publications/i/item/9789240074323 (Accessed June 10, 2024).

[ref74] Zamboanga B. L. Audley S. Olthuis J. V. Blumenthal H. Tomaso C. C. Bui N. . (2019). Validation of a seven-factor structure for the motives for playing drinking games measure. Assessment 26, 582–603. doi: 10.1177/1073191117701191, 28412835 PMC5623648

[ref75] Zhang M. X. Pesigan I. J. A. Kahler C. W. Yip M. C. W. Yu S. Wu A. M. S. (2019). Psychometric properties of a Chinese version of the brief young adult alcohol consequences questionnaire (B-YAACQ). Addict. Behav. 90, 389–394. doi: 10.1016/j.addbeh.2018.11.045, 30529995

