# Supplementary material for: Evaluating alcohol consumption in adolescents and young adults: a meta-analysis of the psychometric properties of measurement instruments
Source: Front Psychol. 2026 Jan 9;16:1591800. doi: 10.3389/fpsyg.2025.1591800 (PMC12827126; doi:10.3389/fpsyg.2025.1591800)
Supplement: Supplementary file 1 [file Table_1.docx]

**Evaluating alcohol consumption in adolescents and young adults: A meta-analysis of psychometric tools**

Supplementary Material

Table of Contents

**Supplementary S1:** Table of search terms used for systematic review

**Supplementary S2:** Figure illustrating scientific output over the past five years

**Supplementary S3.** Psychometric Quality and Risk of Bias Evaluation (Terwee–COSMIN Criteria)

**Supplementary S4:** Table of documented psychometric instruments used in alcohol assessment

**Supplementary S5.** Instrument-specific reliability and model-fit analysis

**Supplementary S6:** Global sensitivity analysis of Cronbach’s alpha

**Supplementary S1.** Table of search terms used for systematic review

| Database | Search terms | n |  |
| --- | --- | --- | --- |
| Scopus | | TITLE-ABS-KEY ("Alcoholism" OR "Alcohol Drinking" OR "Binge Drinking" OR "Underage Drinking" ) AND TITLE-ABS-KEY ( "Child" OR "Adolescent" OR "Young Adult" ) AND TITLE-ABS-KEY ( "Assessment" OR "Measure" OR "Measurement" OR "Diagnosis" ) AND TITLE-ABS-KEY ( "Test" OR "Scale" OR "Questionnaire" ) AND PUBYEAR > 2018 AND PUBYEAR < 2025 AND ( LIMIT-TO ( DOCTYPE , " Article" ) ) | 1980 |
| Web Of Science | ("Alcoholism" OR "Alcohol Drinking" OR "Binge Drinking" OR "Underage Drinking") AND ("Child" OR "Adolescent" OR "Young Adult") AND ("Assessment" OR "Measure" OR "Measurement" OR "Diagnosis") AND ("Test" OR "Scale" OR "Questionnaire") (Topic) and 2019 or 2020 or 2021 or 2022 or 2023 or 2024 (Publication Years) and Article (Document Types) | 1019 |  |
| PsyINFO | ("Alcoholism" OR "Alcohol Drinking" OR "Binge Drinking" OR "Underage Drinking") AND ("Child" OR "Adolescent" OR "Young Adult") AND ("Assessment" OR "Measure" OR "Measurement" OR "Diagnosis") AND ("Test" OR "Scale" OR "Questionnaire") AND (2020 OR 2024) AND Article (“quantitative study”) | 812 |  |
| Pubmed | ("Alcoholism" OR "Alcohol Drinking" OR "Binge Drinking" OR "Underage Drinking") AND ("Child" OR "Adolescent" OR "Young Adult") AND ("Assessment" OR "Measure" OR "Measurement" OR "Diagnosis") AND ("Test" OR "Scale" OR "Questionnaire") Filters: from 2019 - 2024 Sort by: Publication Date | 601 |  |
| Total |  | 4412 |  |

In the PubMed and PsycINFO databases, the search was conducted across "all fields," allowing for the simultaneous identification of specific terms in titles, abstracts, and full texts. In Scopus, the search was performed within the title, abstract, and author-specified keywords fields. In Web of Science, the search was conducted within the "topic" field, which includes titles, abstracts, and indexed keywords.

To ensure comprehensive coverage of the literature and account for differences in database structures and indexing methods, the search string was customized for each database. The following filters were applied to the search results: Document type: “Article” in PsycINFO and Scopus, and “Academic publication” in Web of Science. The Boolean operators "AND" and "OR" were used across all four databases to refine and optimize search outcomes.

**Supplementary S2.** Figure illustrating scientific output over the past five years


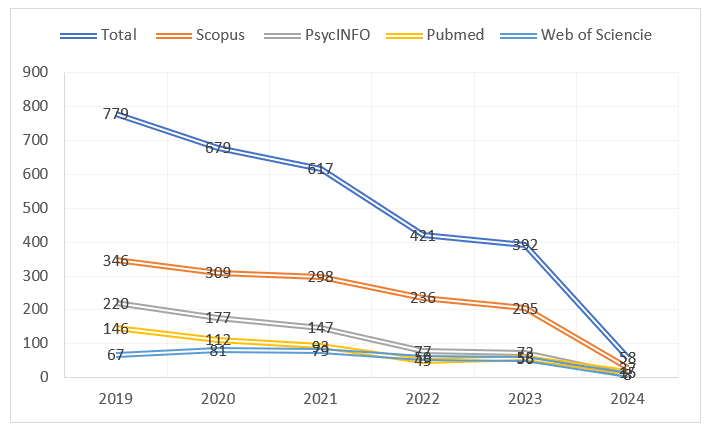


Metrics across the four databases: PubMed, Scopus, Web of Science, and PsycINFO, indicate a notable decline in publications in this field beginning in 2019, with 779 studies. This decline became more pronounced in subsequent years: 679 studies in 2020, 617 in 2021, 421 in 2022, 392 in 2023, and only 58 studies recorded during the first quarter of 2024.

**Supplementary S3.** Psychometric Quality and Risk of Bias Evaluation (Terwee–COSMIN Criteria)

| Instrument | Content validity | Internal  consistency | Criterion validity | Construct validity | Reproducibility/ Agreement | Reproducibility/ Reliability | Responsiveness | Floor and ceiling  effects | Interpretatability |
| --- | --- | --- | --- | --- | --- | --- | --- | --- | --- |
| AUDIT | + | + | + | + | + | + | + | + | + |
| BYAACQ | + | + | + | + | ? | + | + | + | + |
| CLASS | + | + | + | + | ? | + | + | + | + |
| PRQ | + | + | + | + | + | + | + | + | + |
| PDPS | + | + | ? | + | ? | ? | ? | ? | 0 |
| ACQ-SF-R | + | + | 0 | ? | ? | ? | ? | ? | 0 |
| ASQ | + | + | ? | ? | ? | ? | 0 | 0 | 0 |
| SPBSS-20 | + | 0 | ? | ? | ? | ? | 0 | ? | 0 |
| AR2i | ? | + | ? | ? | ? | ? | ? | ? | ? |
| BSCQ | ? | + | ? | ? | ? | ? | ? | ? | ? |
| ABOM | + | + | ? | ? | ? | ? | ? | ? | + |
| Perceived Access to Alcohol and Other Drug Scale | + | + | ? | ? | ? | ? | ? | ? | + |
| TRD | + | + | ? | + | ? | ? | ? | ? | + |
| PARDI | + | + | + | + | ? | ? | ? | ? | + |

**Rating:** + = positive; 0 = intermediate; - = poor;? = no information available.

AUDIT = Alcohol Use Disorders Identification Test (Spanish, English, and Arabic versions); BYAACQ = Brief Young Adult Alcohol Consequences Questionnaire (English, Spanish, Chinese versions); CLASS = College Life Alcohol Salience Scale (English, Spanish versions); PDPS = Protective Drinking Practices Scale (English version); PRQ = Parental rules toward alcohol use (Dutch, English versions); AR2i = (Spanish version); BSCQ = Brief Situational Confidence Questionnaire–Alcohol (English version); ASQ = Alcohol Sensitivity Questionnaire (English version); ABOM = Alcohol-Induced Blackout Measure (English version); Perceived Access to Alcohol and Other Drug Scale (English version); S-PBSS-20 = Protective Behavioral Strategies Scale20 (Spanish version); TRD = Trauma-related drinking to cope (English version); ACQ-SF-R = Alcohol Craving Questionnaire–Short Form–Revised (Portuguese version); PARDI = Responsible Drinking Identity (English version)

| **Section and Topic** | **#** | **Checklist item^a^** | **Location** |
| --- | --- | --- | --- |
| TITLE | | | |
| Title | 1 | Identify the report as a systematic review and include as applicable the following (in any order): outcome domain of interest, population  of interest, name/type of OMIs of interest, and measurement properties of interest. | 1 |
| ABSTRACT | | | |
| OPEN SCIENCE | | | |
| Funding^b^ | 2.2 | Specify the primary source of funding for the review. | 12 |
| Registration | 2.3 | Provide the register name and registration number. | 5 |
| BACKGROUND | | | |
| Objectives | 2.4 | Provide an explicit statement of the main objective(s) or question(s) the review addresses. | 2-3 |
| METHODS | | | |
| Eligibility criteria | 2.5 | Specify the inclusion and exclusion criteria for the review. | 4 |
| Information sources | 2.6 | Specify the information sources (e.g., databases, registers) used to identify studies and the date when each was last searched. | 4 |
| Risk of bias | 2.7 | Specify the methods used to assess risk of bias in the included studies. | 5 |
| Measurement properties | 2.8 | Specify the methods used to rate the results of a measurement property. | 5-6 |
| Synthesis methods | 2.9 | Specify the methods used to present and synthesize results. | 6-7 |
| RESULTS | | | |
| Included studies | 2.10 | Give the total number of included OMIs and study reports. | 6 |
| Synthesis of results | 2.11 | Present the syntheses of results of OMIs, indicating the certainty of the evidence. | 6-8 |
| DISCUSSION | | | |
| Limitations of evidence | 2.12 | Provide a brief summary of the limitations of the evidence included in the review (e.g., study risk of bias, inconsistency, and imprecision). | 10-11 |
| Interpretation | 2.13 | Provide a general interpretation of the results and important implications. | 11-12 |
| PLAIN LANGUAGE SUMMARY | | | |
| Plain language summary | 3 | If allowed by the journal, provide a plain language summary with background information and key findings. | 1 |
| OPEN SCIENCE | | | |
| Registration and protocol | 4a | Provide registration information for the review, including register name and registration number, or state that the review was not registered. | 5 |
|  | 4b | Indicate where the review protocol can be accessed, or state that a protocol was not prepared. | 5 |
|  | 4c | Describe and explain any amendments to information provided at registration or in the protocol. | 5 |
| Support | 5 | Describe sources of financial or non-financial support for the review, and the role of the funders in the review. | 12 |
| Competing interests | 6 | Declare any competing interests of review authors. | 12 |
| Availability of data, code, and other materials | 7 | Report which of the following are publicly available and where they can be found: template data collection forms; data extracted from  included studies; data used for all analyses; analytic code; any other materials used in the review. | 13 |
| INTRODUCTION | | | |
| Rationale | 8 | Describe the rationale for the review in the context of existing knowledge. | 2 |
| Objectives | 9 | Provide an explicit statement of the objective(s) or question(s) the review addresses and include as applicable the following (in any  order): outcome domain of interest, population of interest, name/type of OMIs of interest, and measurement properties of interest. | 2-3 |
| METHODS | | | |
| Followed guidelines | 10 | Specify, with references, the methodology and/or guidelines used to conduct the systematic review. | 5 |
| Eligibility criteria | 11 | Specify the inclusion and exclusion criteria for the review. | 4 |
| Information sources | 12 | Specify all databases, registers, preprint servers, websites, organizations, reference lists and other sources searched or consulted to  identify studies. Specify the date when each source was last searched or consulted. | 4 |
| Search strategy | 13 | Present the full search strategies for all databases, registers, and websites, including any filters and limits used. | 4 |
| Selection process | 14 | Specify the methods used to decide whether a study met the inclusion criteria of the review, e.g., including how many reviewers  screened each record and each report retrieved, whether they worked independently, and if applicable, details of automation tools/AI  used in the process. | 4-5 |
| Data collection process | 15 | Specify the methods used to collect data from reports, e.g., including how many reviewers collected data from each report, whether  they worked independently, any processes for obtaining or confirming data from study investigators, and if applicable, details of  automation tools/AI used in the process. | 5 |
| Data items | 16 | List and define which data were extracted (e.g., characteristics of study populations and OMIs, measurement properties’ results, and  aspects of feasibility and interpretability). Describe methods used to deal with any missing or unclear information. | 6 |
| Study risk of bias assessment | 17 | Specify the methods used to assess risk of bias in the included studies, e.g., including details of the tool(s) used, how many reviewers  assessed each study and whether they worked independently, and if applicable, details of automation tools/AI used in the process. | 5 |
| Measurement properties | 18 | Specify the methods used to rate the results of a measurement property for each individual study and for the summarized or pooled  results, e.g., including how many reviewers rated each study and whether they worked independently. | 5 |
| Synthesis methods | 19a | Describe the processes used to decide which studies were eligible for each synthesis. | 8 |
|  | 19b | Describe any methods used to synthesize results. | 8 |
|  | 19c | If applicable, describe any methods used to explore possible causes of inconsistency among study results (e.g., subgroup analysis). | 8 |
|  | 19d | If applicable, describe any sensitivity analyses conducted to assess robustness of the synthesized results. | 8 |
| Certainty assessment | 20 | Describe any methods used to assess certainty (or confidence) in the body of evidence. | 8-9 |
| Formulating recommendations | 21 | If appropriate, describe any methods used to formulate recommendations regarding the suitability of OMIs for a particular use. | 12 |
| RESULTS | | | |
| Study selection | 22a | Describe the results of the search and selection process, from the number of records identified in the search to the number of study  reports included in the review, ideally using a flow diagram. If applicable, also report the final number of OMIs included and the number  of study reports relevant to each OMI. [T] | 6-8 |
|  | 22b | Cite study reports that might appear to meet the inclusion criteria, but which were excluded, and explain why they were excluded. | 6-8 |
|  |  |  |  |
| OMI characteristics | 23a | Present characteristics of each included OMI, with appropriate references. [T] | 6-8 |
|  | 23b | If applicable, present interpretability aspects for each included OMI. [T] | 6-8 |
|  | 23c | If applicable, present feasibility aspects for each included OMI. [T] | 6-8 |
| Study characteristics | 24 | Cite each included study report evaluating one or more measurement properties and present its characteristics. [T] | 6-8 |
| Risk of bias in studies | 25 | Present assessments of risk of bias for each included study. [T] | 6-8 |
| Results of individual studies | 26 | For all measurement properties, present for each study: (a) the reported result and (b) the rating against quality criteria, ideally using  structured tables or plots. [T] | 6-8 |
| Results of syntheses | 27a | Present results of all syntheses conducted. For each measurement property of an OMI, present: (a) the summarized or pooled result and  (b) the overall rating against quality criteria. [T] | 6-8 |
|  | 27b | If applicable, present results of all investigations of possible causes of inconsistency among study results. | 6-8 |
|  | 27c | If applicable, present results of all sensitivity analyses conducted to assess the robustness of the synthesized results. | 6-8 |
| Certainty of evidence | 28 | Present assessments of certainty (or confidence) in the body of evidence for each measurement property of an OMI assessed. [T] | 11 |
| Recommendations | 29 | If appropriate, make recommendations for suitable OMIs for a particular use. | 12 |
| DISCUSSION | | | |
| Discussion | 30a | Provide a general interpretation of the results in the context of other evidence. | 9-12 |
|  | 30b | Discuss any limitations of the evidence included in the review. | 9-12 |
|  | 30c | Discuss any limitations of the review processes used. | 9-12 |
|  | 30d | Discuss implications of the results for practice, policy, and future research. | 9-12 |

**Supplementary S4.** Table of documented psychometric instruments used in alcohol assessment

| Instrument | n | % |
| --- | --- | --- |
| Alcohol Use Disorders Identification Test (AUDIT; Saunders et al, 1993) | 7 | 25.9 % |
| Brief Young Adult Alcohol Consequences Questionnaire (BYAACQ; Kahler et al, 2005) | 4 | 14.8 % |
| College Life Alcohol Salience Scale (CLASS; Osberg et al, 2010; Bravo et al, 2018) | 3 | 11.1 % |
| Protective Drinking Practices Scale (PDPS; Martin et al., 2020) | 2 | 7.4 % |
| Parental rules toward alcohol use (PRQ; Van der Vorst et al, 2005; 2006) | 2 | 7.4 % |
| AR2i (Cortés et al, 2017) | 1 | 3.7 % |
| Brief Situational Confidence Questionnaire–Alcohol (BSCQ; Delaney et al, 2020) | 1 | 3.7 % |
| Alcohol Sensitivity Questionnaire (ASQ; O’Neill et al, 2002) | 1 | 3.7 % |
| Alcohol-Induced Blackout Measure (ABOM; Miller et al, 2019) | 1 | 3.7 % |
| Perceived Access to Alcohol and Other Drug Scale (Kuntsche et al, 2008) | 1 | 3.7 % |
| Protective Behavioral Strategies Scale20 (S-PBSS-20; Sánchez-García et al, 2020) | 1 | 3.7 % |
| Trauma-related drinking to cope (TRD; Hawn et al, 2020) | 1 | 3.7 % |
| Alcohol Craving Questionnaire–Short Form–Revised (ACQ-SF-R; Singleton, 1995) | 1 | 3.7 % |
| Responsible Drinking Identity (PARDI; Leary et al, 2023) | 1 | 3.7 % |

The most frequently utilized instruments are the Alcohol Use Disorders Identification Test (AUDIT); Brief Young Adult Alcohol Consequences Questionnaire (BYAACQ).

**Supplementary S5.** Instrument-specific reliability and model-fit analysis

| AUDIT |  | Cronbach’s Alpha | CFI | RMSEA | SRMR | TLI |
| --- | --- | --- | --- | --- | --- | --- |
|  | N | 7 | 3 | 3 | 1 | 2 |
|  | Did not report | 0 | 4 | 4 | 6 | 5 |
|  | Mean | 0.826 | 0.956 | 0.0313 | 0.0238 | 0.972 |
|  | Median | 0.800 | 0.976 | 0.410 | 0.0238 | 0.972 |
|  | Standard deviation | 0.0678 | 0.0399 | 0.0185 | NaN | 0.00636 |
|  | Minimum | 0.790 | 0.910 | 0.0100 | 0.0238 | 0.967 |
|  | Maximum | 0.978 | 0.982 | 0.0430 | 0.238 | 0.976 |
| BYAACQ |  | Cronbach’s Alpha | CFI | RMSEA | SRMR | TLI |
|  | N | 4 | 1 | 1 | 0 | 1 |
|  | Did not report | 0 | 3 | 3 | 4 | 3 |
|  | Mean | 0.902 | 0.916 | 0.0550 | NaN | 0.908 |
|  | Median | 0.895 | 0.916 | 0.0550 | NaN | 0.908 |
|  | Standard deviation | 0.0263 | NaN | NaN | NaN | NaN |
|  | Minimum | 0.880 | 0.916 | 0.0550 | NaN | 0.908 |
|  | Maximum | 0.940 | 0.916 | 0.0550 | NaN | 0.908 |
| CLASS |  | Cronbach’s Alpha | CFI | RMSEA | SRMR | TLI |
|  | N | 6 | 6 | 6 | 3 | 3 |
|  | Did not report | 0 | 0 | 0 | 3 | 3 |
|  | Mean | 0.887 | 0.943 | 0.0513 | 0.0423 | 0.938 |
|  | Median | 0.890 | 0.948 | 0.0490 | 0.0400 | 0.952 |
|  | Standard deviation | 0.0344 | 0.0145 | 0.0227 | 0.00874 | 0.00242 |
|  | Minimum | 0.840 | 0.920 | 0.0310 | 0.0350 | 0.910 |
|  | Maximum | 0.920 | 0.955 | 0.0800 | 0.0520 | 0.952 |
| PRQ |  | Cronbach’s Alpha | CFI | RMSEA | SRMR | TLI |
|  | N | 7 | 7 | 7 | 7 | 7 |
|  | Did not report | 0 | 0 | 0 | 0 | 0 |
|  | Mean | 0.893 | 0.940 | 0.0870 | 0.0397 | 0.887 |
|  | Median | 0.890 | 0.950 | 0.0530 | 0.0300 | 0.905 |
|  | Standard deviation | 0.0198 | 0.0262 | 0.0579 | 0.0294 | 0.0554 |
|  | Minimum | 0.870 | 0.886 | 0.0310 | 0.0210 | 0.773 |
|  | Maximum | 0.930 | 0.962 | 0.155 | 0.104 | 0.930 |

The most frequently utilized instruments are the Alcohol Use Disorders Identification Test (AUDIT); Brief Young Adult Alcohol Consequences Questionnaire (BYAACQ).

**Supplementary S6.** Global sensitivity analysis of Cronbach’s alpha

In Model 1, the Egger coefficient was -5.301, and I² = 99.65%, representing the initial model.

**Model 1** **(*n=*37)**


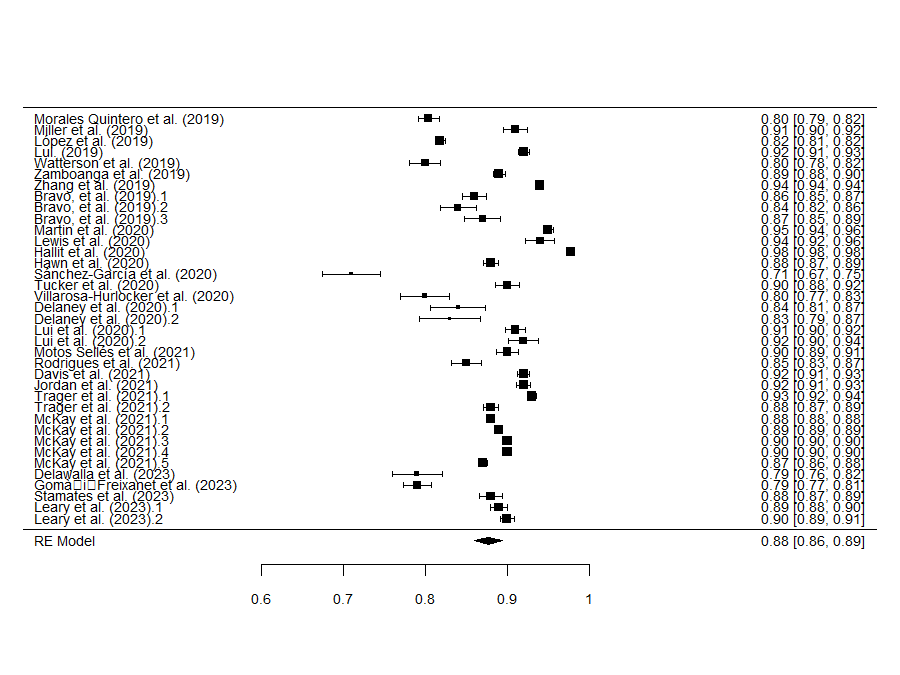


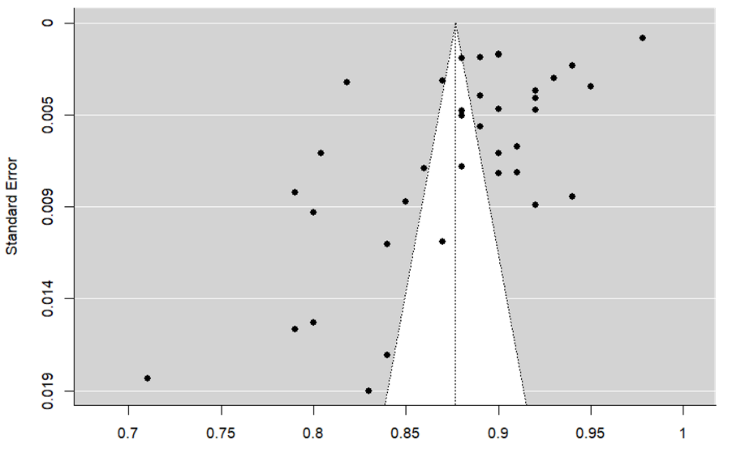


In Model 2, studies with a Cronbach’s alpha lower than 0.80 were excluded. Minor differences were observed in the coefficients, with Egger = -3.317 and I² = 95.5%.

**Model 2 (*n=*34)**


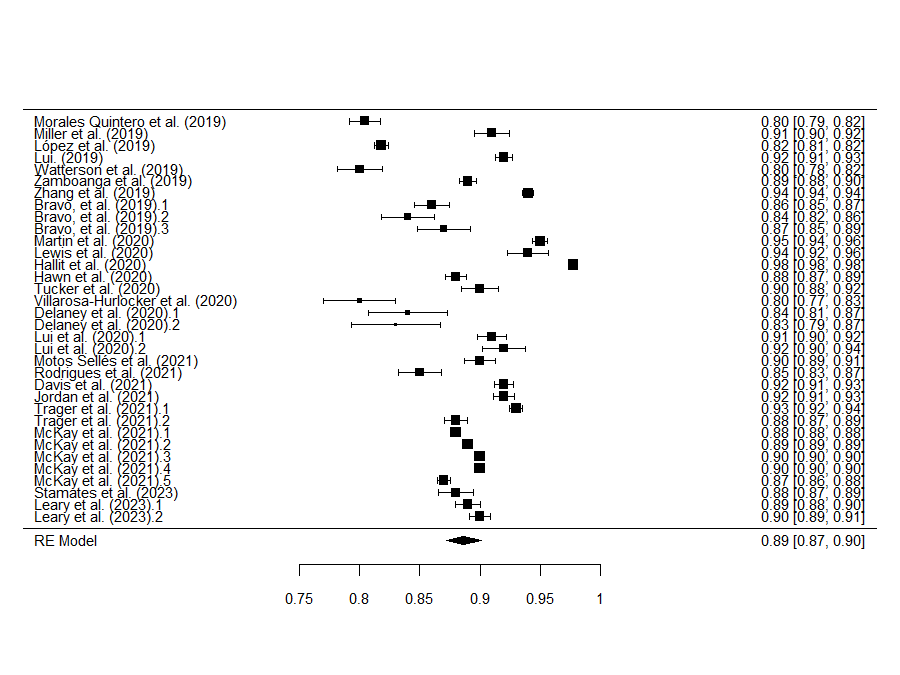


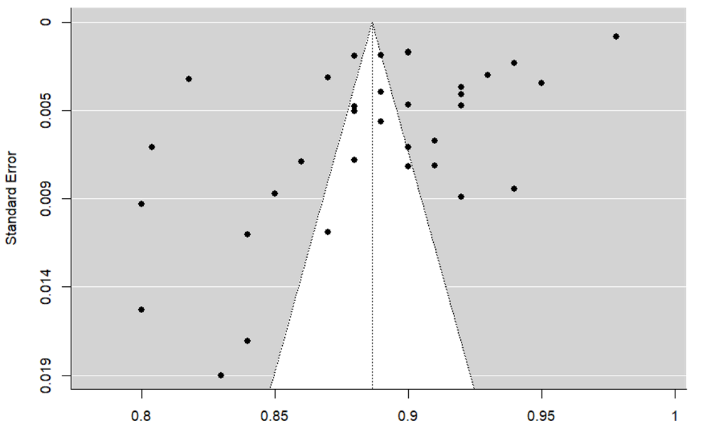


In Model 3, studies with an alpha greater than 0.90 were removed, resulting in Egger = -3.109 and I² = 98.85%.

**Model 3 (n=23)**


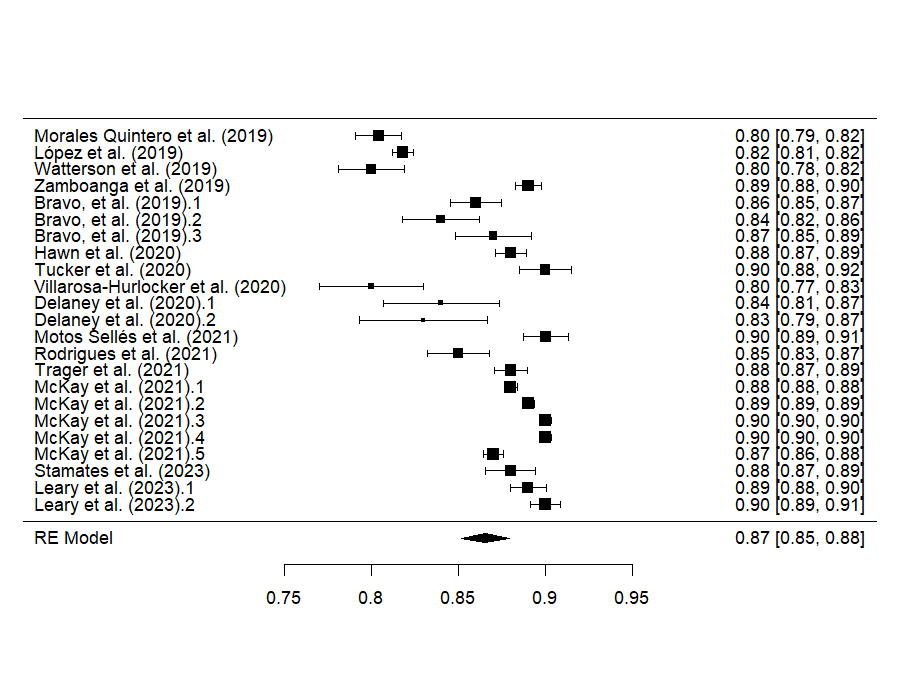


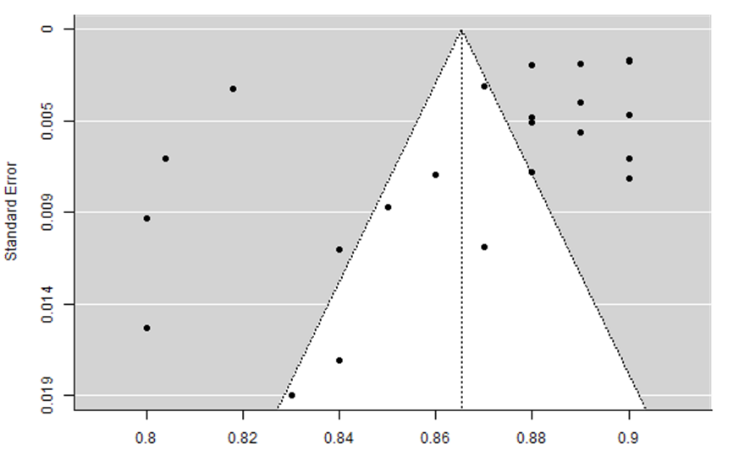


In Model 4, studies with alpha values below 0.83 and above 0.87 were eliminated, yielding Egger = -3.537 and I² = 61.73%.

**Model 4 (n=7)**


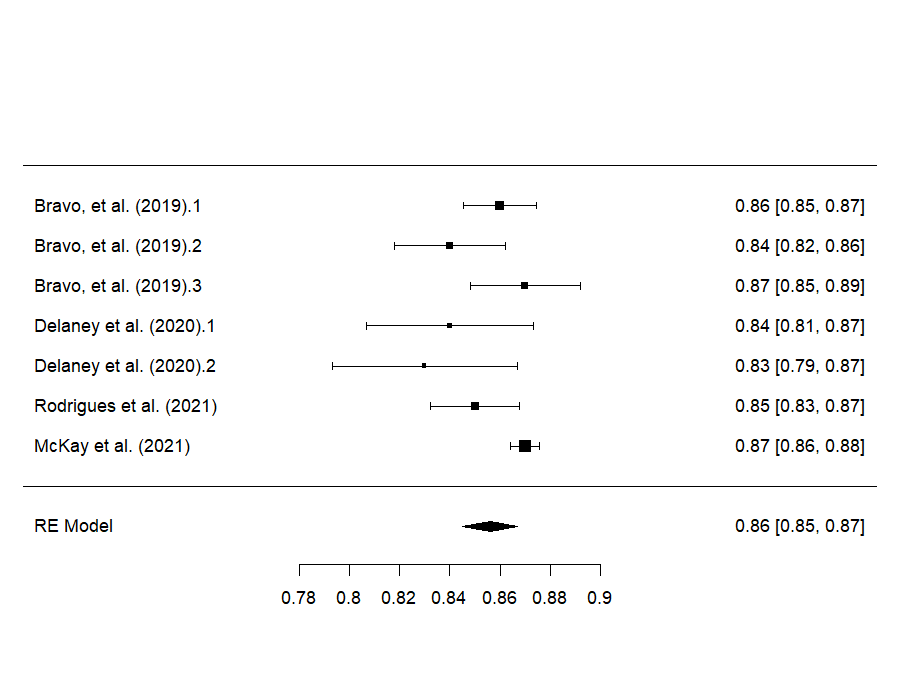


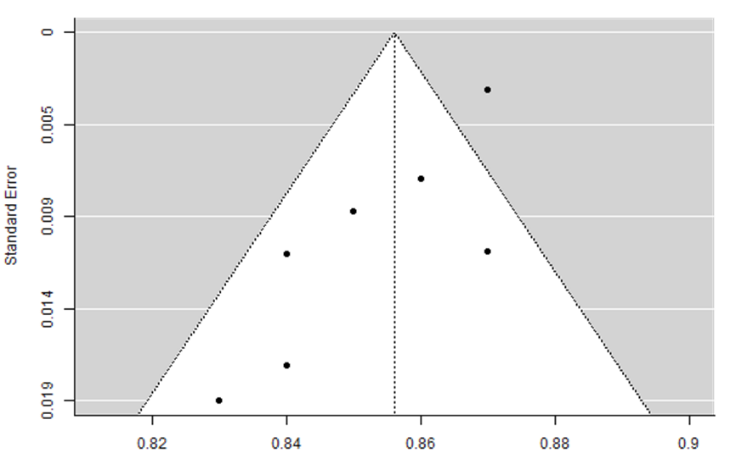


These findings suggest that outliers may influence the initial model.
